# Supplementary material for: Behavioral engagement patterns and psychosocial outcomes in web-based interpretation bias training for anxiety
Source: PLOS Digit Health. 2025 Jul 24;4(7):e0000945. doi: 10.1371/journal.pdig.0000945 (PMC12289040; doi:10.1371/journal.pdig.0000945)
Supplement: S1 File — Supporting information below is available in the online S1 supplement file. Section A NOTES ON MATERIALS AND METHODS – A.1 Excluding Telecoaching Participants– A.2 Cognitive Bias Modification for Interpretation (CBM-I) Details– A.3 Measure Details and Adaptations– A.4 Details for Recognition Ratings– A.5 Details for Brief Body Sensations Interpretations Questionnaire– A.6 Initial Cluster Analysis and Overview of Clustering Algorithms– A.7 Assessment Measures Used for Time-Related Engagement Markers– A.8 Outlier Handling and Transformations Details– A.9 Missing Data Handling for Engagement Markers– A.10 Choosing K-Means Clustering With Two Clusters– A.11 Post Hoc Duda-Hart Test– A.12 Missing Data Handling for Outcome Measures– A.13 Coding of Time– A.14 Search for Auxiliary Variables– A.15 Imputation Model Specifications– A.16 Multilevel Model Specifications Section B TABLES – B.1 Demographic Characteristics by Engagement Group– B.2 Raw Descriptive Statistics of Outcomes by Engagement Group Over Time– B.3 Out-of-Range Scores Across 100 Imputed Datasets– B.4 Post Hoc Tests of Engagement Group Differences at Baseline– B.5 Tests of Group Differences in Engagement Markers for Sensitivity Analysis– B.6 Piecewise Linear Multilevel Modeling Results for Sensitivity Analysis– B.7 Piecewise Linear Multilevel Modeling Simple Time Effects for Significant Interaction Effects for Sensitivity Analysis– B.8 Internal Validation Measures for Different Clustering Algorithms for 2-4 Clusters– B.9 Stability Validation Measures for Different Clustering Algorithms for 2-4 Clusters– B.10 Optimal Number of Clusters Suggested by Clustering Validity Indices– B.11 Number of Days Elapsed Between Consecutive Training Sessions– B.12 Piecewise Linear Multilevel Modeling Random Effects– B.13 Correlations Among Initial Engagement Features Section C FIGURES – C.1 Box and Violin Plots of Time on Training Components by Engagement Group– C.2 Box Plots of the Log of Time on Assessment Measures by Engagemen [file pdig.0000945.s001.pdf]

Supplement S1 for behavioral engagement patterns and psychosocial outcomes in web-based interpretation bias training for anxiety

Ángel Francisco Vela de la Garza Evia[1¶*], Jeremy William Eberle[2,3¶*], Sonia Baee[1], Emma Catherine Wolfe[2], Mehdi Boukhechba[1], Daniel Harold Funk[4], Bethany Ann Teachman[2*], Laura Elizabeth Barnes[1]

[1]Department of Systems and Information Engineering, University of Virginia, Charlottesville, Virginia, USA

[2]Department of Psychology, University of Virginia, Charlottesville, Virginia, USA

[3]Department of Medical Social Sciences, Northwestern University Feinberg School of Medicine, Chicago, Illinois, USA

[4]Sartography, Staunton, Virginia, USA

* Corresponding Author

E-mail: afv9x@virignia.edu (AFV)

E-mail: jwe4ec@virginia.edu (JWE)

E-mail: bteachman@virginia.edu (BAT)

¶These joint first authors contributed equally to this work.

Section A NOTES ON MATERIALS AND METHODS

A.1 Excluding Telecoaching Participants

837 participants who completed the first training session and a few of its measures were classified by a machine learning algorithm as either lower ($n = 288$) or higher ($n = 547$) risk for dropout before starting the second training session. Higher-risk participants were then randomly assigned to either receive supplemental telecoaching ($n = 282$) or continue in only CBM-I ($n = 265$). We excluded the 282 assigned to coaching to focus on participants assigned to only CBM-I (e.g., given that coaching was itself an added intervention to increase engagement and retention).

A.2 Cognitive Bias Modification for Interpretation (CBM-I) Details

In Sessions 1-2 one letter was missing, in Sessions 3-4 two letters were missing, and in Session 5 half of the scenarios had two letters missing and half had the entire word missing. For comprehension questions, participants answered a yes/no question in Sessions 1, 3, and 5 (e.g., "Did your neighbor purposely ignore your call to them in the street?") or chose one of two phrases that completed a sentence about the scenario in Sessions 2 and 4. Participants had multiple chances to complete the word fragment and comprehension question correctly to advance.

A.3 Measure Details and Adaptations

For all measures, numbers were displayed alongside anchors only when the study was completed via computer; numbers were not displayed alongside anchors when the study was completed via mobile devices.

A.3.1 Overall Anxiety Severity and Impairment Scale (OASIS)

To reduce assessment burden, the response options for every item were shortened. For example, for the item assessing frequency of anxiety, the options were *no anxiety* (vs. *No anxiety in the past week.*), *infrequent anxiety* (vs. *Infrequent anxiety. Felt anxious a few times.*), *occasional anxiety* (vs. *Occasional anxiety. Felt anxious as much of the time as not. It was hard to relax.*), *frequent anxiety* (vs. *Frequent anxiety. Felt*

anxious most of the time. It was very difficult to relax.), and *constant anxiety* (vs. *Constant anxiety. Felt anxious all of the time and never really relaxed.*).

We also revised the wording of some response options. For the item assessing avoidance, we used *never* (vs. *None: I do not avoid places, situations, activities, or things because of fear.*). For the two impairment items, we used *not at all* (vs. *None: No interference at work/home/school from anxiety* and *None: My anxiety doesn't affect my relationships.*). For the avoidance and impairment items, we also turned adjectives into adverbs; for the avoidance item we used *infrequently* (vs. *Infrequent...*), *occasionally* (vs. *Occasional...*), and *frequently* (vs. *Frequent...*), and for the impairment items we used *mildly* (vs. *Mild...*), *moderately* (vs. *Moderate...*), *severely* (vs. *Severe...*), and *extremely* (vs. *Extreme...*).

A.3.2 Depression, Anxiety, Stress Scales-Short Form: Anxiety Subscale (DASS-21 AS)

To reduce assessment burden, the response options for every item were shortened. We used *not at all* (vs. *Did not apply to me at all*), *sometimes* (vs. *Applied to me to some degree, or some of the time*), *a lot of the time* (vs. *Applied to me a considerable degree, or a good part of the time*), and *most of the time* (vs. *Applied to me very much, or most of the time*). We also administered the items in a fixed (vs. random) order.

A.3.3 Recognition Ratings (RR)

Although the types of the four possible final sentences for each scenario were not changed from the original (i.e., two representing positive or negative threat-relevant interpretations, two representing positive or negative foil, threat-irrelevant interpretations), the comprehension questions and items for 2 scenarios used in the original were modified, and the other 7 scenarios and their comprehension questions and items were newly created by our team. Specifically, the 9 scenarios we used were selected out of a set of 18 scenarios (6 for each threat type) that were piloted in a sample of 274 participants on Amazon Mechanical Turk in October 2015; the 3 scenarios whose negative threat-related ratings most highly correlated with anxiety (total score on OASIS) within each threat type were selected. See Supplement S2 at https://osf.io/wynxs/ for the full measure and its differences from the original.

A.3.4 Brief Body Sensations Interpretation Questionnaire (BBSIQ)

The ranking task in the original was excluded. For ratings, instead of the original 9-point Likert scale ranging from 0 (*not at all likely*) to 8 (*extremely likely*), with intermediate anchors at 2 (*a little*), 4 (*moderately*), and 6 (*very*), we used a 5-point Likert scale ranging from 0 (*not at all likely*) to 4 (*extremely likely*), with the same intermediate anchors placed at 1, 2, and 3. The wording of some scenarios and explanations was also modified to reflect American (vs. British) English. See Supplement S2 at https://osf.io/wynxs/ for the full measure and its differences from the original.

A.4 Details for Recognition Ratings

Three scenarios are about social threats, three are about physical/health threats, and three are about other threats. An example scenario is "The Loud Noise: You are woken up in the middle of the night by a loud noise. You are not sure what caused the noise and leave your bedroom to see what happened. You walk... downst_irs." After imagining themselves in the scenario and correctly completing the last word, a neutral word fragment (e.g., selecting "a" to complete "downstairs"), participants answered a reading comprehension question that retained the scenario's ambiguity (e.g., "Have you been woken up in the middle of the night?"). Following the comprehension questions, for each scenario, participants saw the title (e.g., THE LOUD NOISE), a starting sentence (e.g., "As you walk downstairs..."), and the four disambiguated interpretations. Of the four interpretations, two were threat related (negative: "You feel afraid, and worry that you cannot handle the fear."; positive: "You feel afraid, but you know that you can tolerate the feeling.") and the other two were threat unrelated (negative: "You feel cold, and think about how the house needs better heating."; positive: "You feel happy, and think about how lovely your house is.").

A.5 Details for Brief Body Sensations Interpretations Questionnaire

Seven scenarios are about panic body sensations; seven are about social or other events. An example scenario is "A friend suggests that you change the way that you're doing a job in your own house. Why?" For each situation, three potential explanations are provided. One explanation is always negative and threat relevant (e.g., "They think you're incompetent."); the other two are mixed in valence but not threat relevant (e.g.,

“They are trying to be helpful.” and “They have done the job more often and know an easier way.”).

A.6 Initial Cluster Analysis and Overview of Clustering Algorithms

A.6.1 Initial Cluster Analysis

We were interested in grouping participants based on engagement markers to create engagement groups. Before clustering, we visualized the histogram of the time-related engagement marker features and noticed that most had a right-skewed distribution due to outliers. Hence, we log-transformed these features to get their distributions closer to normal and proceeded to standardize all of the engagement marker features, including completion rate. Then, we started off by including task completion rate but fewer time-related markers and found two clusters differing in completion rate, similar to the partitioning in other papers. [1] However, given that most participants in the group with the lower completion rate did not complete the first training session and that training and assessment tasks were administered in series (e.g., Session 1 assessment is not administered until Session 1 training is completed; vs. in parallel), such participants had assessment data only at baseline. The lack of assessment data at subsequent time points led to nonconvergence of the imputation model used to handle missing outcome data. Thus, we ultimately included additional time-related markers, resulting in two groups (see Section A.10) differing in time spent that each had enough assessment data for the imputation model to converge.

A.6.2 Overview of Clustering Algorithms

Clustering is an unsupervised learning method commonly used as an exploratory classification technique to suggest unknown subgroups in data. To do the clustering we evaluated three algorithms: K-means, partitioning around medoids (PAM), and agglomerative hierarchical clustering. For K-means clustering, the number of clusters K needs to be specified at the start of the analysis to then partition the data into K distinct, non-overlapping clusters. This partitioning minimizes the total within-cluster variation (defined using squared Euclidean distance). K-means clustering is done in two iterative steps. First, the algorithm is initiated by randomly assigning all observations to a number from 1 to K. Then, the centroid is calculated for the K clusters, and observations are reassigned to the cluster with the nearest centroid. This second step

is repeated until the observation assignments no longer change, indicating that the model has converged. [2] The K-medoids algorithm, PAM, tries to minimize the sum of dissimilarities between observations making it more robust than K-means to noise and outliers. [3] Instead of calculating the centroids for each cluster as in K-means, actual data points are selected as medoids. A medoid is a data point in a cluster that has the lowest average dissimilarity to the rest of the other points. The PAM algorithm consists of the build and swap phases. K points are randomly initiated in the build phase as medoids and observations are assigned to the cluster with the closest medoid. A different non-medoid data point is randomly selected as a medoid in the swap phase and swapped with the initial medoid. If the swapping minimizes the objective function, then a new set of medoids are defined, and observations are reassigned. This process is repeated until the medoids stop changing. [4] For both of these clustering methods, we used the Euclidean distance.

Agglomerative hierarchical clustering is another common clustering technique. Each observation starts as an individual cluster, and the pairwise inter-cluster dissimilarities are calculated using a distance metric such as Euclidean distance. Then, the algorithm proceeds to merge the two clusters with the highest similarity. The new pairwise inter-cluster dissimilarities are calculated for the rest of the other clusters based on the linkage criterion, which is a function of the distance metric. This is repeated until all of the observations fall under one single cluster. [2] Finally, the outcome is visualized in a dendrogram. To extract the clusters, one must specify at what height to cut the dendrogram. It is important to note that different distance metrics and linkage methods yield varying clustering results. For our analysis, we used Euclidean distance and Ward's linkage.

A.7 Assessment Measures Used for Time-Related Engagement Markers

The one-time measures included demographic variables, mental health history, anxiety triggers, importance of reducing anxiety (Importance Ruler, modified from original) [5], training confidence (Readiness Ruler), and frequency of device use (measures without citations were developed by our team).

The repeated measures included measures of interpretation bias (RR, BBSIQ); anxiety symptoms (OASIS, DASS-21 AS); comorbid depression symptoms (Patient Health Questionnaire-2, PHQ-2, adapted from original) [6] and alcohol use (Alcohol Use Disorders Identification Test-Concise, AUDIT-C, modified from

original) [7]; centrality of anxiety to identity (Anxiety and Identity Circles, modified from original) [8]; other cognitive mechanisms, including cognitive flexibility (Cognitive Flexibility Inventory, CFI, adapted from original) [9], experiential avoidance (Comprehensive Assessment of ACT Processes, CompACT, modified from original) [10], cognitive reappraisal (Emotion Regulation Questionnaire, ERQ, modified from original) [11], and intolerance of uncertainty (Intolerance of Uncertainty Scale-Short Form, IUS-12, modified from original) [12]; and wellness, including self-efficacy (New General Self-Efficacy Scale, NGSES, modified from original) [13], growth mindset (Personal Beliefs Survey, PBS, modified from original) [14], optimism (Life Orientation Test-Revised, LOT-R, modified from original) [15], and life satisfaction; [16] and state anxiety (Subjective Units of Distress, SUDS, modified from original) [17].

A.8 Outlier Handling and Transformations Details

We defined an outlier as a value three median absolute deviations from the median [18] and handled outliers for time spent on CBM-I scenarios separate from outliers for other time-related engagement markers. For time spent on scenarios, we identified scenarios with outlying time spent values within a given participant's session and excluded these scenarios (approximately 10%) when computing average time spent per CBM-I scenario across sessions. A boxplot visualization also showed that one participant completed only two scenarios and spent more than 20 min on them; we treated this participant as if they had done no scenarios.

For other time-related engagement markers, we computed the proportion of such markers with outlying values for each participant and excluded two participants from all analyses because more than 70% of their markers were outliers. We then reduced the impact of outlying time-related markers by capping extreme values; [19] specifically, for all of these markers, values in the bottom 1% were replaced with the value of the 1st percentile, and values in the top 1% were replaced with the value of the 99th percentile. Next, we log-transformed these markers given that histograms revealed that most still had right-skewed distributions. Finally, we standardized all markers, including completion rate, before conducting the cluster analysis.

A.9 Missing Data Handling for Engagement Markers

A monotone missing data pattern for time-related engagement markers resulted from attrition; participants who withdrew or did not return did not complete remaining training or assessment tasks. In these cases, we used the mean of the available time values. If a participant had no time values for a marker (e.g., completed no CBM-I scenarios), we treated the marker (e.g., mean time per scenario) as zero.

A.10 Choosing K-Means Clustering With Two Clusters

A.10.1 Choosing K-Means Clustering

To choose the clustering algorithm (out of K-means, PAM, and agglomerative hierarchical clustering), we computed validation indices and visualized the resulting clusters.

Specifically, we used the `clValid` package (ver. 0.7) [20] to compute internal and stability validation measures for the three algorithms with two to four clusters. For internal validation measures, we computed connectivity, silhouette width, and the Dunn index. These measures account for the compactness, connectedness, and separation of the clusters. [20] Connectivity values should be minimized, whereas silhouette width and Dunn index values should be maximized. [20] For stability validation measures, we computed average proportion of non-overlap (APN), average distance (AD), average distance between means (ADM), and figure of merit (FOM). These measures compare the results from clustering with all the features versus the results from clustering by excluding one feature at each iteration. All of these measures should be minimized. [20] For internal validation measures, see Table B.8. For stability validation measures, see Table B.9. We discuss the relevant results of these measures below, after discussing our visualization of the clusters.

For visualization, we ran the `fviz_cluster` function of the `factoextra` package (ver. 1.0.7) [21] to visualize the clusters in two dimensions (i.e., using the first two principal components from principal components analysis). Visually inspecting Fig C.6, we noticed that the three algorithms with four clusters and the agglomerative hierarchical clustering algorithm with three clusters each produced a group of participants (i.e., the bottommost group in each relevant plot) that resembled a group of participants that had led to convergence issues in our initial cluster analysis (see Section A.6.1). Specifically, most of the participants

in this group did not complete the first training session, meaning that they had outcome data only at baseline. The lack of subsequent outcome data for such participants in our initial cluster analysis led to nonconvergence in the imputation model used to handle missing outcome data. Therefore, we discarded these options (the three algorithms with four clusters and the agglomerative hierarchical clustering algorithm with three clusters) to avoid running into this issue again. When inspecting the three algorithms with two clusters, we also noticed that agglomerative hierarchical clustering created clusters that were more imbalanced (ns = 268 and 429) than those created by K-means (ns = 386 and 311) and PAM (ns = 360 and 337), so we also discarded that option, which removed agglomerative hierarchical clustering from consideration entirely.

Comparing internal and stability validation measures (see Table B.8 and Table B.9) for K-means and PAM for two and three clusters (the remaining options), K-means had superior scores on more measures than PAM. Specifically, for two and three clusters, respectively, K-means had lower connectivity (161.4321, 152.5024), APN (0.0460, 0.0890), AD (4.6604, 4.4384), and ADM (0.1920, 0.3546) scores, and a higher silhouette score (0.2457, 0.2578), whereas PAM had only a lower FOM score (0.8596, 0.8245) and a higher Dunn score (0.1099, 0.1145). Although both algorithms yielded similar clusters based on visual inspection of Fig C.6, we chose K-means given its superior validation measures and greater popularity. [22–24]

A.10.2 Choosing Two Clusters

Once we selected the K-means algorithm, we used the `NbClust` package (ver. 3.0) [22] to compute clustering validity indices to determine the optimal number of clusters for K-means based on majority rule. After running this package for 2-10 clusters, we interpreted only the 23 numerical indices computed, out of which the majority (9) of the indices proposed two clusters as the optimal number of clusters (see Table B.10).

A.10.3 Final Cluster Methodology

Our final two clusters were created with the `kmeans` function of the `stats` package (ver. 4.3.1) [25], using the Lloyd algorithm, two centers, and 30 iterations as parameters.

A.11 Post Hoc Duda-Hart Test

We also ran a Duda-Hart homogeneity test [26] post hoc with the `dudahart2` function of the `fpc` package (ver. 2.2-12). [27] This test compares the same ratio ($SSE_2/SST = .71$) to a critical value (.94 for our sample size, number of features, and α set to .05 following another study [28]) of an approximate asymptotic distribution of the ratio under a multivariate normal null distribution. [26, 29] Although the test was significant ($p < .001$), supporting two clusters, simulations have shown that when features have non-negligible intercorrelations (rs $> .1$) and the sample size and number of features are similar to ours, K-means with the Duda-Hart test tends to support two clusters even under a multivariate normal data-generating distribution. [28] By contrast, another simulation study found that the LBR test had a Type I error rate of 0, even with highly intercorrelated features (rs $= .8$). [30] We focus on the LBR as the more conservative test given that our features still had non-negligible correlations after excluding features that intercorrelated at least .70 from the cluster analysis (see Table B.13).

A.12 Missing Data Handling for Outcome Measures

We had two patterns of missing data for outcome measures. A general missing data pattern at the item level resulted from endorsements of "prefer not to answer." However, this was infrequent; across the five outcomes, the proportion of incomplete item responses ranged from 0.10% to 0.62%. In these cases, scale scores were computed as the mean of available items.

A monotone missing data pattern at the scale level resulted from attrition. The proportions of missing scale scores across the outcomes ranged from 55.7% to 57.6% (see Table B.2). We imputed missing scale scores using fully Bayesian model-based multiple imputation [31] in `Blimp` (ver. 1.3.6). [32] For details, see Section A.15.

A.13 Coding of Time

For the OASIS outcome, which was assessed at all timepoints, time_{TR} and time_{FU} were coded as described in the main text. For the other outcomes, which were assessed at only four timepoints, time_{TR} was coded as 0 for baseline, as 3 for Session 3, as 5 for Session 5, and as 5 for follow-up; time_{FU} was coded as 0 for

baseline, Session 3, and Session 5 and as 1 for follow-up.

A.14 Search for Auxiliary Variables

To identify auxiliary variables to include in the imputation analysis, we analyzed differences in the mean proportion of missing assessments on categorical demographic variables. [33] We included these variables as auxiliary variables in the imputation model if their mean proportion difference between two levels with more than 100 participants was greater than .1. Device used throughout the intervention (one device vs. multiple devices) and gender (male vs. female) were the only variables that met these criteria. Device used had a mean proportion difference greater than .2. Gender's mean proportion difference was greater than .1; a one-way ANOVA confirmed that this difference was significant. We included these two variables as auxiliary variables and assumed that data are missing at random for the imputation and multilevel models.

In the imputation model, gender was collapsed into male, female, and transgender/other, with "prefer not to answer" responses treated as missing values; device used was collapsed into one device (e.g., desktop only) and multiple devices (e.g., mobile and desktop) and was then refactored into a binary variable (one device or not) to aid model convergence.

A.15 Imputation Model Specifications

We built a separate imputation model congenial with our substantive analysis model for each outcome. [34] Given that a search for auxiliary variables (see Section A.14) had found that gender and device used throughout the study related to missingness, we also included these variables in the imputation model. We imputed 100 datasets for each outcome.

Each imputation model included the fixed effects of engagement group, time_{TR}, time_{FU}, Engagement Group $\times$ Time_{TR}, Engagement Group $\times$ Time_{FU}, training confidence (grand mean centered), and the auxiliary variables gender and device used. We also included a random intercept and random slopes for time_{TR} and time_{FU}. Codings for engagement group, time_{TR}, and time_{FU} were the same as those used in the substantive analysis model. For the sensitivity analysis, `Blimp` used more time spent as the reference group, but for the multilevel model analysis less time spent was used as the reference group to be consistent

with the main analysis.

In `Blimp`, we specified device used as ordinal and gender and engagement groups as nominal. Training confidence, treated as continuous, was grand mean centered (using the `grandmean` argument).

By study and analysis design, assessment point, engagement group, and device used were complete; therefore, we specified these variables as fixed to aid model convergence. [35] Training confidence was not specified as a fixed variable since it had missing values.

Imputations were saved every 5,000 iterations after the burn-in period, which varied between outcomes and ranged from 10,000 to 100,000 burn-in iterations (OASIS: 10,000; DASS-21 AS: 20,000; BBSIQ: 50,000; RR Negative Bias: 100,000; RR Positive Bias: 50,000). These burn-in iterations were selected based on convergence diagnosis. We diagnosed convergence by checking that the split-chain potential scale reduction factor was less than 1.05 at the final burn-in interval and that the effective number of Markov chain Monte Carlo (MCMC) samples was greater than 100. [35]

We used the default software imputation configurations: two MCMC chains with random starting values, homogeneous within-cluster variances, and priors for the dependent (`prior2`) and predictor (`xprior2`) variables. [35]

Given that the imputation models produced values outside the permitted scale range, we assessed the mean percentage of out-of-range values for each imputed dataset (see Table B.3). For any scale at a given timepoint, the mean percentage did not go over 12%. We considered these values not to be large enough to inflate variance estimates. [36]

A.16 Multilevel Model Specifications

Because `Blimp` imputes data in each variable's original metric (even when centering variables in the imputation model), training confidence was grand mean centered in each imputed dataset prior to analysis.

Each model was fit by maximizing the restricted log-likelihood. In order to handle convergence errors we adjusted some of the control parameters in `lmeControl`. [37,38] For the OASIS, DASS21, BBSIQ, and RR Positive Bias multilevel models, we switched from the default `nlminb` optimizer to the `optim` optimizer. For the RR Negative Bias model we used the `optim` optimizer, increased the maximum number of iterations for

the optimization algorithm (`msMaxIter = 1e9`), and increased the number of iterations for the expected maximization algorithm (`niterEM = 1000`).

When pooling results, we adjusted the `df.com` parameter in the `testEstimates` function with Barnard and Rubin's (1999) procedure to cap the degrees of freedom at values below those had we worked with complete data.

Section B TABLES

Table B.1: Demographic Characteristics by Engagement Group

Characteristic	Analyzed Sample (n = 697)	Less Time Spent (n = 386)	More Time Spent (n = 311)
**Age (years)**: M (SD)	35.38 (11.9)	31.94 (9.6)	39.66 (13.0)
**Gender**: n (%)			
Female	564 (80.9)	316 (81.9)	248 (79.7)
Male	116 (16.6)	59 (15.3)	57 (18.3)
Transgender [a]	2 (0.3)	1 (0.3)	1 (0.3)
Transgender Female [a]	0 (0)	0 (0)	0 (0)
Transgender Male [a]	3 (0.4)	2 (0.5)	1 (0.3)
Other	10 (1.4)	7 (1.8)	3 (1)
Prefer not to answer	2 (0.3)	1 (0.3)	1 (0.3)
**Race**: n (%)			
American Indian/Alaska Native	7 (1)	2 (0.5)	5 (1.6)
Black/African origin	69 (9.9)	33 (8.5)	36 (11.6)
East Asian	15 (2.2)	12 (3.1)	3 (1)
Native Hawaiian/Pacific Islander	4 (0.6)	1 (0.3)	3 (1)
South Asian	13 (1.9)	9 (2.3)	4 (1.3)
White/European origin	491 (70.4)	270 (69.9)	221 (71.1)
More than one race	58 (8.3)	41 (10.6)	17 (5.5)
Other or Unknown	32 (4.6)	13 (3.4)	19 (6.1)
Prefer not to answer	8 (1.1)	5 (1.3)	3 (1)
**Ethnicity**: n (%)			
Hispanic or Latino	93 (13.3)	48 (12.4)	45 (14.5)
Not Hispanic or Latino	570 (81.8)	325 (84.2)	245 (78.8)
Unknown	14 (2)	6 (1.6)	8 (2.6)
Prefer not to answer	20 (2.9)	7 (1.8)	13 (4.2)
**Country**: n (%)			
United States	639 (91.7)	367 (95.1)	272 (87.5)
Australia	28 (4)	8 (2.1)	20 (6.4)
Canada	8 (1.1)	3 (0.8)	5 (1.6)
United Kingdom	8 (1.1)	5 (1.3)	3 (1)
Other	13 [b](1.9)	3 (0.8)	10 (3.2)
Prefer not to answer	1 (0.1)	0 (0)	1 (0.3)
**Education**: n (%)			
Junior High	1 (0.1)	1 (0.3)	0 (0)
Some High School	9 (1.3)	5 (1.3)	4 (1.3)
High School Graduate	59 (8.5)	30 (7.8)	29 (9.3)
Some College	225 (32.3)	112 (29)	113 (36.3)
Associate's Degree	75 (10.8)	45 (11.7)	30 (9.6)
Bachelor's Degree	164 (23.5)	90 (23.3)	74 (23.8)
Some Graduate School	38 (5.5)	27 (7)	11 (3.5)
Master's Degree	84 (12.1)	53 (13.7)	31 (10)
M.B.A.	13 (1.9)	10 (2.6)	3 (1)
J.D.	3 (0.4)	0 (0)	3 (1)
M.D.	2 (0.3)	1 (0.3)	1 (0.3)
Ph.D.	10 (1.4)	6 (1.6)	4 (1.3)
Other Advanced Degree	12 (1.7)	6 (1.6)	6 (1.9)

Prefer not to answer	2 (0.3)	0 (0)	2 (0.6)
**Employment Status**: n (%)			
Student	96 (13.8)	65 (16.8)	31 (10)
Homemaker	60 (8.6)	29 (7.5)	31 (10)
Unemployed or laid off	33 (4.7)	13 (3.4)	20 (6.4)
Looking for work	38 (5.5)	19 (4.9)	19 (6.1)
Working part-time	107 (15.4)	56 (14.5)	51 (16.4)
Working full-time	295 (42.3)	182 (47.2)	113 (36.3)
Retired	22 (3.2)	4 (1)	18 (5.8)
Other	42 (6)	16 (4.1)	26 (8.4)
Unknown	0 (0)	0 (0)	0 (0)
Prefer not to answer	4 (0.6)	2 (0.5)	2 (0.6)
**Annual Income**: n (%)			
Less than $5,000	33 (4.7)	13 (3.4)	20 (6.4)
$5,000 through $11,999	52 (7.5)	25 (6.5)	27 (8.7)
$12,000 through $15,999	27 (3.9)	15 (3.9)	12 (3.9)
$16,000 through $24,999	63 (9)	32 (8.3)	31 (10)
$25,000 through $34,999	67 (9.6)	36 (9.3)	31 (10)
$35,000 through $49,999	102 (14.6)	58 (15)	44 (14.1)
$50,000 through $74,999	111 (15.9)	68 (17.6)	43 (13.8)
$75,000 through $99,999	68 (9.8)	45 (11.7)	23 (7.4)
$100,000 through $149,999	67 (9.6)	42 (10.9)	25 (8)
$150,000 through $199,999	18 (2.6)	10 (2.6)	8 (2.6)
$200,000 through $249,999	11 (1.6)	5 (1.3)	6 (1.9)
$250,000 or greater	12 (1.7)	9 (2.3)	3 (1)
Unknown	28 (4)	16 (4.1)	12 (3.9)
Prefer not to answer	38 (5.5)	12 (3.1)	26 (8.4)
**Marital Status**: n (%)			
Single	187 (26.8)	103 (26.7)	84 (27)
Dating	83 (11.9)	48 (12.4)	35 (11.3)
Engaged	33 (4.7)	22 (5.7)	11 (3.5)
In a marriage-like relationship	88 (12.6)	51 (13.2)	37 (11.9)
Married	203 (29.1)	117 (30.3)	86 (27.7)
In a domestic/civil union	25 (3.6)	12 (3.1)	13 (4.2)
Separated	16 (2.3)	7 (1.8)	9 (2.9)
Divorced	45 (6.5)	21 (5.4)	24 (7.7)
Widow/widower	7 (1)	1 (0.3)	6 (1.9)
Other	6 (0.9)	3 (0.8)	3 (1)
Prefer not to answer	4 (0.6)	1 (0.3)	3 (1)

[a] Partway through data collection (on 8/5/2019), Transgender was replaced by Transgender Female and Transgender Male.
[b] Collapsed into Other: Germany (n = 2), India (n = 2), Ireland (n = 2), Colombia (n = 1), Croatia (n = 1), Ecuador (n = 1), Jordan (n = 1), Malaysia (n = 1), South Africa (n = 1), United Arab Emirates (n = 1).

Table B.2: Raw Descriptive Statistics of Outcomes by Engagement Group Over Time

Outcome	Timepoint	Analyzed Sample			Less Time Spent			More Time Spent		
		n	*M*	*SD*	*n*	*M*	*SD*	*n*	*M*	*SD*
OASIS	Baseline [a]	697	2.31	0.70	386	2.28	0.71	311	2.34	0.69
	Session 1	549	2.19	0.74	288	2.18	0.74	261	2.20	0.73
	Session 2	397	1.74	0.74	219	1.72	0.74	178	1.76	0.73
	Session 3	356	1.76	0.79	199	1.79	0.82	157	1.73	0.76
	Session 4	295	1.56	0.82	164	1.56	0.81	131	1.56	0.84
	Session 5	272	1.54	0.78	152	1.58	0.77	120	1.50	0.78
	Follow-Up	244	1.57	0.83	140	1.59	0.81	104	1.54	0.86
DASS-21 AS	Baseline [b]	697	1.61	0.54	386	1.65	0.54	311	1.56	0.53
	Session 3	355	1.00	0.61	198	1.06	0.62	157	0.92	0.59
	Session 5	272	0.86	0.57	152	0.95	0.56	120	0.75	0.57
	Follow-Up	244	0.81	0.60	140	0.87	0.59	104	0.73	0.60
BBSIQ	Baseline [a]	697	1.54	0.84	386	1.59	0.84	311	1.46	0.83
	Session 3	347	0.89	0.73	195	0.93	0.74	152	0.84	0.72
	Session 5	270	0.79	0.68	151	0.83	0.71	119	0.74	0.63
	Follow-Up	240 [c]	0.79	0.62	137	0.88	0.66	103	0.66	0.54
RR Negative Bias	Baseline [a]	696 [d]	2.90	0.53	386	2.95	0.54	310	2.83	0.51
	Session 3	352	2.53	0.55	197	2.52	0.57	155	2.53	0.54
	Session 5	270	2.55	0.55	151	2.56	0.57	119	2.55	0.54
	Follow-Up	241	2.52	0.55	137	2.57	0.54	104	2.45	0.56
RR Positive Bias	Baseline [a]	696 [d]	2.31	0.53	386	2.31	0.52	310	2.31	0.53
	Session 3	351	2.87	0.46	196	2.86	0.48	155	2.88	0.44
	Session 5	270	2.87	0.48	151	2.88	0.45	119	2.86	0.52
	Follow-Up	241	2.75	0.47	137	2.77	0.47	104	2.73	0.47

Note. OASIS = Overall Anxiety Severity and Impairment Scale; DASS-21 AS = Depression, Anxiety, Stress Scales-Short Form: Anxiety Subscale; BBSIQ = Brief Body Sensations Interpretations Questionnaire; RR = Recognition Ratings

[a] Assessed during pretreatment.

[b] Assessed during eligibility screener.

[c] One participant endorsed "prefer not to answer" for all BBSIQ items at Follow-Up.

[d] One participant endorsed "prefer not to answer" for all RR items at baseline.

Table B.3: Out-of-Range Scores Across 100 Imputed Datasets

				Minimum Score			Maximum Score	
Outome	Timepoint	Possible Range	*M* % Below	*M*	Absolute	*M* % Above	*M*	Absolute
OASIS	Baseline [a]	[0, 4]	0.00	0.00	0.00	0.00	4.00	4.00
	Session 1		0.06	0.00	-0.82	0.11	4.16	5.00
	Session 2		0.16	-0.25	-0.98	0.19	4.27	5.02
	Session 3		0.42	-0.45	-1.43	0.16	4.21	4.98
	Session 4		1.06	-0.66	-1.53	0.15	4.18	4.89
	Session 5		2.47	-1.01	-1.91	0.16	4.16	5.08
	Follow-up		1.81	-0.93	-2.32	0.21	4.28	5.30
DASS-21 AS	Baseline [b]	[0, 3]	0.00	0.57	0.57	0.00	3.00	3.00
	Session 3		0.95	-0.46	-1.34	0.06	3.05	3.70
	Session 5		5.57	-0.97	-1.41	0.03	2.82	3.40
	Follow-up		5.35	-0.96	-1.91	0.03	2.92	3.33
BBSIQ	Baseline [a]	[0, 4]	0.00	0.00	0.00	0.00	4.00	4.00
	Session 3		3.59	-0.96	-1.78	0.00	4.00	4.00
	Session 5		11.10	-1.53	-2.42	0.00	3.93	3.99
	Follow-up		7.64	-1.12	-1.97	0.00	3.12	4.06
RR Negative Bias	Baseline [a]	[1, 4]	0.00	1.33	1.33	0.00	4.00	4.01
	Session 3		0.08	0.94	0.34	0.21	4.16	4.80
	Session 5		0.55	0.60	0.00	0.29	4.24	4.83
	Follow-up		0.29	0.76	0.15	0.29	4.22	4.96
RR Positive Bias	Baseline [a]	[1, 4]	0.00	1.00	1.00	0.00	4.00	4.00
	Session 3		0.01	0.99	0.85	0.18	4.14	4.51
	Session 5		0.01	1.25	0.57	1.82	4.59	5.54
	Follow-up		0.01	1.26	0.75	0.41	4.25	4.87

Note. OASIS = Overall Anxiety Severity and Impairment Scale; DASS-21 AS = Depression, Anxiety, Stress Scales-Short Form: Anxiety Subscale; BBSIQ = Brief Body Sensations Interpretations Questionnaire; RR = Recognition Ratings

[a] Assessed during pretreatment.

[b] Assessed during eligibility screener.

Table B.4: Post Hoc Tests of Engagement Group Differences at Baseline

Variable	Test	Statistic	p	r	Cramér's V
**Demographic Characteristics**					
Age	Wilcoxon rank-sum test	38,719.0	<.001***	.30	
Gender	Chi-squared test	1.8	.401		.05
Race	Chi-squared test [a]	18.2	.011*		.16
Ethnicity	Chi-squared test	0.7	.389		.03
Education	Wilcoxon rank-sum test	64,702.0	.042*	.08	
**Mental Health**					
OASIS	Wilcoxon rank-sum test	57,164.0	.278	.04	
DASS-21 AS	Wilcoxon rank-sum test	66,004.0	.023*	.09	
BBSIQ	Wilcoxon rank-sum test	65,731.5	.031*	.08	
RR Negative Bias	Wilcoxon rank-sum test	68,202.0	.001**	.12	
RR Positive Bias	Wilcoxon rank-sum test	60,695.0	.742	.01	
PHQ-2	Wilcoxon rank-sum test	56,019.5	.199	.05	
AUDIT-C	Wilcoxon rank-sum test	59,921.0	.649	.02	
**Training Confidence**					
Training Confidence	Wilcoxon rank-sum test	58,773.0	.598	.02	

Note. OASIS = Overall Anxiety Severity and Impairment Scale; DASS-21 AS = Depression, Anxiety, Stress Scales-Short Form: Anxiety Subscale; BBSIQ = Brief Body Sensations Interpretations Questionnaire; RR = Recognition Ratings; PHQ = Patient Health Questionnaire; AUDIT-C = Alcohol Use Disorders Identification Test. *$p < .05$. **$p < .01$. ***$p < .001$. For the categorical demographic characteristics we performed the following changes due to small numbers of participants in some categories. For gender, race, ethnicity, and education we changed "Prefer not to answer" to "NA" (treated as missing); we also recoded "Unknown" as "NA" for ethnicity and education. For gender we collapsed "Transgender", "Transgender Female", "Transgender Male", and "Other" to "Transgender/Other", resulting in the categories "Female", "Male", and "Transgender/Other". For education, we collapsed "Junior High" and "Some High School" to "Not High School Graduate"; "Associate's Degree" and "Some College" to "Some College"; "Bachelor's Degree" to "College Degree"; and "J.D.", "M.B.A.", "M.D.", "Master's Degree","Other Advanced Degree", and "Ph.D." to "Advanced Degree"; this resulted in the categories "Not High School Graduate", "Some College", "College Degree", "Some Graduate School", and "Advanced Degree". Per Cohen (1988), rs and Cramér's Vs $\geq$ .1, .3, or .5 are small, medium, or large, respectively. [39]

[a] Due to small expected counts (< 5) in some categories of race, we also ran a Fisher's exact test using the `fisher.test` function of the `stats` package, which similarly yielded a significant result (p = .009).

Table B.5: Tests of Group Differences in Engagement Markers for Sensitivity Analysis

Engagement Marker	Less Time Spent (n = 323) Median (IQR)	More Time Spent (n = 304) Median (IQR)	Wilcoxon rank-sum test	p
**Task completion rate**	0.57 (0.69)	0.57 (0.69)	46,284.0	0.198
**Time on training components**				
Time on imagery practice exercise (min)	0.91 (0.57)	1.46 (0.90)	75,114.0	<.001
Time on anxiety imagery prime exercise (min)	1.71 (0.73)	2.59 (1.46)	80,391.0	<.001
Mean time per CBM-I scenario across sessions (sec)	11.75 (6.03)	14.25 (5.48)	63,019.0	<.001
**Mean time on assessment tasks (min)**				
Anxiety (DASS-21 AS)[a]	0.52 (0.20)	0.81 (0.41)	84,987.5	<.001
Credibility	0.28 (0.35)	0.83 (0.64)	79,708.5	<.001
Demographics	1.05 (0.38)	1.72 (0.77)	85,924.5	<.001
Mental Health History	1.07 (0.48)	1.95 (1.08)	87,454.5	<.001
Anxiety Identity[a]	0.19 (0.09)	0.32 (0.16)	82,739.0	<.001
Anxiety (OASIS)[a]	0.44 (0.16)	0.71 (0.29)	88,532.0	<.001
Anxiety Triggers	0.65 (0.34)	1.21 (0.67)	84,850.0	<.001
Interpretation Bias (RR)[a]	2.53 (0.84)	4.11 (1.52)	89,451.5	<.001
Depression and Alc. Use (Comorbid)[a]	0.43 (0.19)	0.70 (0.28)	84,425.5	<.001
Wellness[a]	0.69 (0.27)	1.21 (0.47)	90,709.5	<.001
Mechanisms[a]	0.54 (0.24)	0.88 (0.41)	85,799.5	<.001
Technology Use	0.25 (0.11)	0.39 (0.19)	81,334.5	<.001
Affect[a]	0.12 (0.06)	0.21 (0.12)	81,844.0	<.001

Note. Time spent on the Brief Body Sensations Interpretations Questionnaire was excluded given its high correlation with time spent on RR, Mechanisms, and Wellness measures. IQR = interquartile range; DASS-21 AS = Depression, Anxiety, Stress Scales-Short Form: Anxiety Subscale; OASIS = Overall Anxiety Severity and Impairment Scale; RR = Recognition Ratings. For measure details, see Table 1.

[a] Repeated measure.

Table B.6: Piecewise Linear Multilevel Modeling Results for Sensitivity Analysis

Outcome	Fixed Effect	b (SE)	t	df	p	95% CI	Random Effect	s^2	r		
									1	2	3
OASIS	Intercept	2.24(0.04)	57.56	3,442.81	<.001***	[2.17, 2.32]	1. Intercept	0.38	-		
	$time_{TR}$	-0.15(0.01)	-14.90	641.02	<.001***	[-0.17, -0.13]	2. $time_{TR}$	0.01	-0.18	-	
	$time_{FU}$	0.13(0.06)	2.07	322.20	.039*	[0.01, 0.25]	3. $time_{FU}$	0.33	-0.13	-0.40	-
	More Time Spent	0.09(0.06)	1.54	592.42	.124	[-0.02, 0.20]	Residual	0.18			
	Training confidence	-0.02(0.03)	-0.47	541.09	.636	[-0.08, 0.05]					
	More Time Spent × $time_{TR}$	-0.01(0.02)	-0.85	451.63	.396	[-0.04, 0.02]					
	More Time Spent × $time_{FU}$	0.00(0.09)	0.00	257.35	.996	[-0.19, 0.19]					
DASS21	Intercept	1.63(0.03)	54.71	1,810.74	<.001***	[1.58, 1.69]	1. Intercept	0.17	-		
	$time_{TR}$	-0.15(0.01)	-16.91	332.66	<.001***	[-0.17, -0.13]	2. $time_{TR}$	0.00	0.00	-	
	$time_{FU}$	-0.02(0.04)	-0.57	292.33	.568	[-0.10, 0.05]	3. $time_{FU}$	0.04	-0.49	0.09	-
	More Time Spent	-0.09(0.04)	-2.08	601.31	.038*	[-0.17, -0.00]	Residual	0.12			
	Training confidence	-0.02(0.03)	-0.61	515.15	.543	[-0.06, 0.03]					
	More Time Spent × $time_{TR}$	-0.01(0.01)	-1.05	334.57	.294	[-0.04, 0.01]					
	More Time Spent × $time_{FU}$	0.08(0.06)	1.33	214.86	.184	[-0.04, 0.20]					
BBSIQ	Intercept	1.58(0.05)	34.40	1,839.45	<.001***	[1.49, 1.67]	1. Intercept	0.52	-		
	$time_{TR}$	-0.17(0.01)	-13.32	442.06	<.001***	[-0.20, -0.15]	2. $time_{TR}$	0.02	-0.63	-	
	$time_{FU}$	0.17(0.05)	3.44	213.17	.001**	[0.07, 0.27]	3. $time_{FU}$	0.03	0.06	-0.75	-
	More Time Spent	-0.12(0.07)	-1.78	611.80	.075	[-0.25, 0.01]	Residual	0.18			
	Training confidence	0.02(0.03)	0.72	377.75	.471	[-0.04, 0.09]					
	More Time Spent × $time_{TR}$	0.01(0.02)	0.75	368.25	.455	[-0.02, 0.05]					
	More Time Spent × $time_{FU}$	-0.16(0.07)	-2.25	218.13	.026*	[-0.30, -0.02]					
RR Negative Bias	Intercept	2.95(0.03)	102.50	1,819.56	<.001***	[2.89, 3.00]	1. Intercept	0.14	-		
	$time_{TR}$	-0.10(0.01)	-10.79	417.39	<.001***	[-0.12, -0.08]	2. $time_{TR}$	0.01	-0.26	-	
	$time_{FU}$	0.14(0.04)	3.33	223.86	.001**	[0.06, 0.22]	3. $time_{FU}$	0.01	-0.22	-0.46	-
	More Time Spent	-0.13(0.04)	-3.27	607.85	.001**	[-0.22, -0.05]	Residual	0.14			
	Training confidence	0.00(0.02)	0.16	439.96	.872	[-0.04, 0.05]					
	More Time Spent × $time_{TR}$	0.03(0.01)	2.21	347.87	.028*	[0.00, 0.06]					
	More Time Spent × $time_{FU}$	-0.17(0.06)	-2.73	220.90	.007**	[-0.29, -0.05]					
RR Positive Bias	Intercept	2.33(0.03)	80.89	1,809.56	<.001***	[2.28, 2.39]	1. Intercept	0.14	-		
	$time_{TR}$	0.13(0.01)	14.13	570.93	<.001***	[0.11, 0.15]	2. $time_{TR}$	0.01	-0.60	-	
	$time_{FU}$	-0.23(0.04)	-5.37	233.33	<.001***	[-0.32, -0.15]	3. $time_{FU}$	0.03	0.47	-0.86	-
	More Time Spent	-0.01(0.04)	-0.28	601.89	.778	[-0.09, 0.07]	Residual	0.14			
	Training confidence	0.01(0.02)	0.54	311.89	.586	[-0.03, 0.05]					
	More Time Spent × $time_{TR}$	0.01(0.01)	0.45	461.46	.654	[-0.02, 0.03]					

More Time Spent × $time_{FU}$	-0.01(0.07)	-0.16	195.62	.873	[-0.14, 0.12]

Note. Each outcome was modeled separately. Every model had the fixed effects of engagement group, $time_{TR}$, $time_{FU}$, engagement group × $time_{TR}$, engagement group × $time_{FU}$, training confidence (grand mean centered), a random intercept, and random slopes for $time_{TR}$ and $time_{FU}$. Engagement group was dummy coded with Less Time Spent as the reference group (0 = Less Time Spent, 1 = More Time Spent). OASIS = Overall Anxiety Severity and Impairment Scale; DASS-21 AS = Depression, Anxiety, Stress Scales-Short Form: Anxiety Subscale; BBSIQ = Brief Body Sensations Interpretations Questionnaire; RR = Recognition Ratings; TR = training trajectory; FU = follow-up trajectory. *$p < .05$. **$p < .01$. ***$p < .001$.

Table B.7: Piecewise Linear Multilevel Modeling Simple Time Effects for Significant Interaction Effects for Sensitivity Analysis

Outcome	*Fixed Effect*	*b (SE)*	*t*	*df*	*p*	*95% CI*
BBSIQ	More Time Spent × $time_{FU}$	-0.16(0.07)	-2.25	218.13	.026*	[-0.30, -0.02]
	$Time_{FU(LessTimeSpent)}$	0.17(0.05)	3.41	174.03	.001**	[0.07, 0.27]
	$Time_{FU(MoreTimeSpent)}$	0.01(0.05)	0.22	148.59	.824	[-0.09, 0.12]
RR Negative Bias	More Time Spent × $time_{TR}$	0.03(0.01)	2.21	347.87	.028*	[0.00, 0.06]
	$Time_{TR(LessTimeSpent)}$	-0.10(0.01)	-10.43	338.06	<.001***	[-0.12, -0.08]
	$Time_{TR(MoreTimeSpent)}$	-0.07(0.01)	-7.04	215.51	<.001***	[-0.09, -0.05]
	More Time Spent × $time_{FU}$	-0.17(0.06)	-2.73	220.90	.007**	[-0.29, -0.05]
	$Time_{FU(LessTimeSpent)}$	0.14(0.04)	3.29	185.92	.001**	[0.06, 0.22]
	$Time_{FU(MoreTimeSpent)}$	-0.03(0.04)	-0.59	158.71	.558	[-0.11, 0.06]

Note. For the simple time effects, separate models were fit for each engagement group with fixed effects for $time_{TR}$, $time_{FU}$, and training confidence (grand mean centered), a random intercept, and random slopes for $Time_{TR}$ and $Time_{FU}$. Simple time effects were analyzed only for significant ($p < 0.05$) interactions in Table B.6. BBSIQ = Brief Body Sensations Interpretations Questionnaire; RR = Recognition Ratings; TR = training trajectory; FU = follow-up trajectory. *$p < .05$. **$p < .01$. ***$p < .001$.

Table B.8: Internal Validation Measures for Different Clustering Algorithms for 2-4 Clusters

Algorithm	Measure	2	3	4
K-means	Connectivity	161.4321	152.5024	285.1087
	Dunn	0.1081	0.1012	0.0998
	Silhouette	0.2457	0.2578	0.1742
Partitioning Around Medoids	Connectivity	172.4746	318.8563	303.9690
	Dunn	0.1099	0.1145	0.1037
	Silhouette	0.2427	0.1438	0.1616
Agglomerative Hierarchical Clustering	Connectivity	155.7341	156.6532	261.6817
	Dunn	0.0991	0.1139	0.1139
	Silhouette	0.2276	0.2266	0.1473

Note. Indices were computed with the `clValid` package (ver. 0.7). [20] Connectivity values should be minimized, whereas silhouette width and Dunn index values should be maximized. [20]

Table B.9: Stability Validation Measures for Different Clustering Algorithms for 2-4 Clusters

Algorithm	Measure	2	3	4
K-means	APN	0.0460	0.0890	0.1846
	AD	4.6604	4.4384	4.3393
	ADM	0.1920	0.3546	1.0029
	FOM	0.8615	0.8450	0.8041
Partitioning Around Medoids	APN	0.0630	0.2046	0.2540
	AD	4.6886	4.5328	4.3625
	ADM	0.2739	0.7486	0.9805
	FOM	0.8596	0.8245	0.8073
Agglomerative Hierarchical Clustering	APN	0.0038	0.0122	0.0222
	AD	5.4252	5.2269	5.2234
	ADM	0.0315	0.0978	0.1667
	FOM	0.9801	0.9661	0.9591

Note. Indices were computed with the `clValid` package (ver. 0.7). [20]. APN = average proportion of non-overlap; AD = average distance; ADM = average distance between means; FOM = figure of merit. All of these measures should be minimized. [20]

Table B.10: Optimal Number of Clusters Suggested by Clustering Validity Indices

Index	Optimal Number of Clusters	Index Value
KL (Krzanowski & Lai)	9	7.68
CH (Calinski & Harabasz)	2	287.75
Hartigan	4	62.13
CCC (Cubic Clustering Criterion)	9	3.72
Scott	4	1108.71
Marriot	4	6.97e+43
TrCovW (Trace of Within Cluster Covariance Matrix)	4	122197.50
TraceW (Trace of W)	4	601.96
Friedman	4	7.19
Rubin	4	-0.12
Cindex	10	0.20
DB (Davies-Bouldin)	2	1.55
Silhouette	2	0.25
Duda	2	1.09
PseudoT2	2	-39.32
Beale	2	-0.93
Ratkowsky	2	0.36
Ball	3	1708.68
PtBiserial	4	0.48
McClain	2	0.74
Dunn	9	0.11
SDindex	2	1.33
SDbw	10	0.571

Note. Numerical validity indices for 2-10 clusters from the K-means algorithm were computed with the `NbClust` package (ver. 3.0). [22] The optimal number of clusters suggested by each index and the index's value for that number of clusters are shown. Out of all 23 indices, 9 indices (the majority) proposed 2 clusters as the optimal number of clusters. By contrast, 8 indices proposed 4 clusters as the optimal number, 3 indices proposed 9 clusters as the optimal number, 2 indices proposed 10 clusters as the optimal number, and 1 index proposed 3 clusters as the optimal number.

Table B.11: Number of Days Elapsed Between Consecutive Training Sessions

Statistic	Session 1-2 ($n = 399$)	Session 2-3 ($n = 366$)	Session 3-4 ($n = 295$)	Session 4-5 ($n = 277$)
Minimum	4.87	4.79	4.91	4.87
1st Quartile	7.26	7.23	7.75	7.67
Median	8.68	8.23	9.33	8.88
Mean	10.98	9.93	11.53	10.56
3rd Quartile	12.35	11.20	13.75	12.34
Maximum	44.83	23.63	40.37	29.81

Note. Numbers of days elapsed between the last task observed for a given session (e.g., Session 1) and the first task observed for the next session (e.g., Session 2) are shown for 699 participants, before excluding the 2 participants who had outlying values in most of the engagement markers. The sample size for each column reflects participants who completed at least one task in the latter session within each pair of sessions. Extreme values were capped within each column; the bottom 1% were set to the 1st percentile, and the top 1% were set to the 99th percentile.

Table B.12: Piecewise Linear Multilevel Modeling Random Effects

			r		
Outcome	Random Effect	s^2	1	2	3
OASIS	1. Intercept	0.38	—		
	2. time_{TR}	0.01	-.18	—	
	3. time_{FU}	0.34	-.14	-.40	—
	Residual	0.18			
DASS-21 AS	1. Intercept	0.17	—		
	2. time_{TR}	0.00	.00	—	
	3. time_{FU}	0.04	-.46	.11	—
	Residual	0.12			
BBSIQ	1. Intercept	0.52	—		
	2. time_{TR}	0.02	-.63	—	
	3. time_{FU}	0.03	.03	-.71	—
	Residual	0.18			
RR Negative Bias	1. Intercept	0.14	—		
	2. time_{TR}	0.01	-.25	—	
	3. time_{FU}	0.01	-.20	-.38	—
	Residual	0.14			
RR Positive Bias	1. Intercept	0.14	—		
	2. time_{TR}	0.01	-.57	—	
	3. time_{FU}	0.03	.44	-.85	—
	Residual	0.13			

Note. Each outcome was modeled separately. Every model had the fixed effects of engagement group, time_{TR}, time_{FU}, engagement group × time_{TR}, engagement group × time_{FU}, training confidence (grand mean centered), a random intercept, and random slopes for time_{TR} and time_{FU} (for fixed effects, see Table 3). Engagement group was dummy coded with Less Time Spent as the reference group (0 = Less Time Spent, 1 = More Time Spent). OASIS = Overall Anxiety Severity and Impairment Scale; DASS-21 AS = Depression, Anxiety, Stress Scales-Short Form: Anxiety Subscale; BBSIQ = Brief Body Sensations Interpretations Questionnaire; RR = Recognition Ratings; TR = training trajectory; FU = follow-up trajectory.

Table B.13: Correlations Among Initial Engagement Features

	Task Completion Rate	Mean Time Per CBM-I Scenario Across Sessions	Time on Imagery Practice Exercise	Time on Anxiety Imagery Prime Exercise	Time on Anxiety Identity	Time on Anxiety Triggers	Time on Interpretation Bias (BBSIQ)	Time on Depression and Alc. Use (Comorbid)	Time on Credibility	Time on Anxiety Symptoms (DASS-21 AS)	Time on Demographics	Time on Mechanisms	Time on Mental Health History	Time on Anxiety Symptoms (OASIS)	Time on Affect	Time on Interpretation Bias (RR)	Time on Technology Use	Time on Wellness
**Task Completion Rate**	—	.40	.12	.17	-.16	.05	-.16	-.09	.12	-.21	.03	-.12	.06	-.09	-.09	-.19	-.02	-.12
**Mean Time Per CBM-I Scenario Across Sessions**	.40	—	.25	.32	.02	.12	.09	.12	.16	.07	.19	.11	.18	.15	.15	.10	.07	.15
**Time on Imagery Practice Exercise**	.12	.25	—	.38	.22	.28	.31	.30	.29	.24	.27	.28	.30	.28	.21	.28	.26	.31
**Time on Anxiety Imagery Prime Exercise**	.17	.32	.38	—	.18	.26	.25	.27	.30	.25	.24	.24	.31	.25	.24	.30	.21	.27
**Time on Anxiety Identity**	-.16	.02	.22	.18	—	.43	.55	.47	.27	.50	.40	.45	.44	.52	.36	.55	.39	.48
**Time on Anxiety Triggers**	.05	.12	.28	.26	.43	—	.56	.47	.54	.49	.53	.49	.58	.58	.35	.53	.43	.53
**Time on Interpretation Bias (BBSIQ)**	-.16	.09	.31	.25	.55	.56	—	.65	.41	.58	.51	**.71**	.51	.65	.45	**.77**	.57	**.75**
**Time on Depression and Alc. Use (Comorbid)**	-.09	.12	.30	.27	.47	.47	.65	—	.36	.46	.43	.59	.46	.51	.44	.56	.45	.61
**Time on Credibility**	.12	.16	.29	.30	.27	.54	.41	.36	—	.41	.43	.36	.42	.42	.26	.44	.35	.41
**Time on Anxiety Symptoms (DASS-21 AS)**	-.21	.07	.24	.25	.50	.49	.58	.46	.41	—	.47	.49	.51	.58	.35	.57	.40	.59
**Time on Demographics**	.03	.19	.27	.24	.40	.53	.51	.43	.43	.47	—	.44	.58	.55	.37	.54	.48	.50
**Time on Mechanisms**	-.12	.11	.28	.24	.45	.49	**.71**	.59	.36	.49	.44	—	.41	.57	.36	.60	.46	.68
**Time on Mental Health History**	.06	.18	.30	.31	.44	.58	.51	.46	.42	.51	.44	.41	—	.54	.39	.50	.44	.51
**Time on Anxiety Symptoms (OASIS)**	-.09	.15	.28	.25	.52	.58	.65	.51	.42	.58	.55	.57	.54	—	.39	.60	.52	.64
**Time on Affect**	-.09	.15	.21	.24	.36	.35	.45	.44	.26	.35	.37	.36	.39	.39	—	.41	.38	.42
**Time on Interpretation Bias (RR)**	-.19	.10	.28	.30	.55	.53	**.77**	.56	.44	.57	.54	.60	.50	.60	.41	—	.49	.65
**Time on Technology Use**	-.02	.07	.26	.21	.39	.43	.57	.45	.35	.40	.48	.46	.44	.52	.38	.49	—	.54
**Time on Wellness**	-.12	.15	.31	.27	.48	.53	**.75**	.61	.41	.59	.50	.68	.51	.64	.42	.65	.54	—

Note. We used the `cor` function of the `stats` package (ver. 4.3.1) [25] to compute Pearson product-moment correlations among the initial engagement features. We excluded features (i.e., time spent on BBSIQ) with high correlations ($\geq .7$), which are in boldface, from the cluster analysis. For the repeated assessment measures (Anxiety Symptoms, Anxiety Identity, Interpretation Bias, Depression and Alc. Use (Comorbid), Wellness, Mechanisms, and Affect), the average time spent was used. For more information about the assessment measures, see Table 1. BBSIQ = Brief Body Sensations Interpretation Questionnaire; RR = Recognition Ratings; OASIS = Overall Anxiety Severity and Impairment Scale; DASS-21 AS = Anxiety Scale of Depression Anxiety Stress Scales.

Section C FIGURES

Fig C.1: Box and Violin Plots of Time on Training Components by Engagement Group

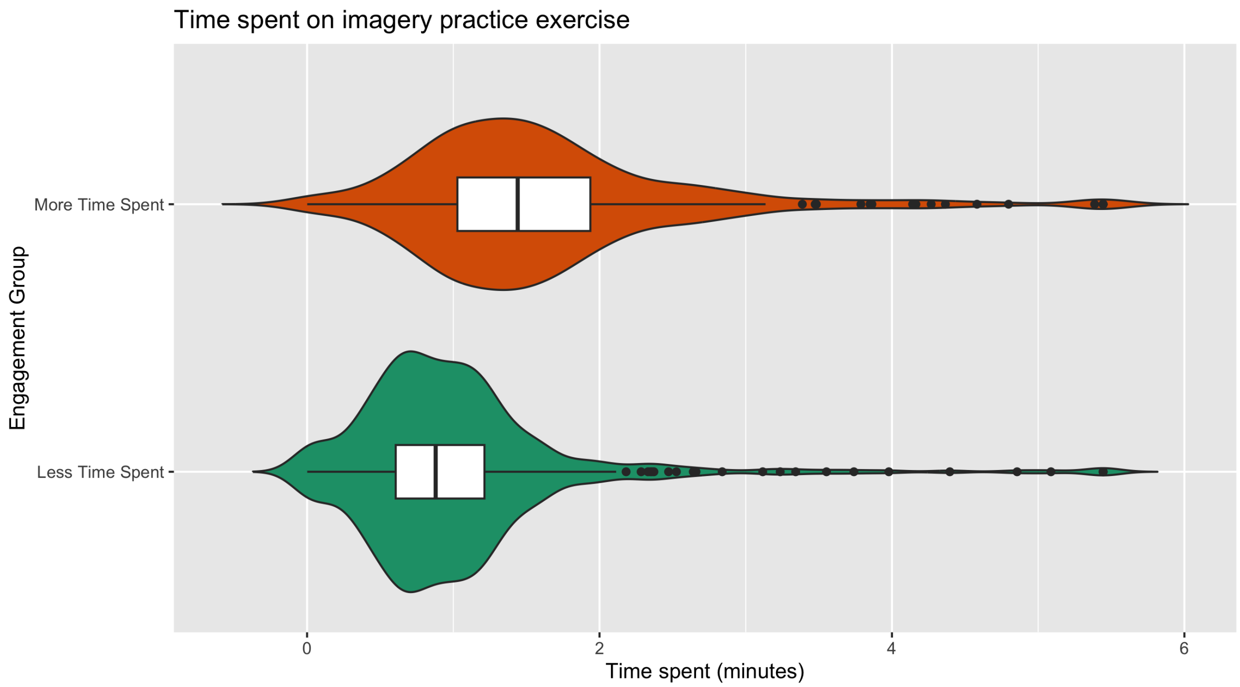

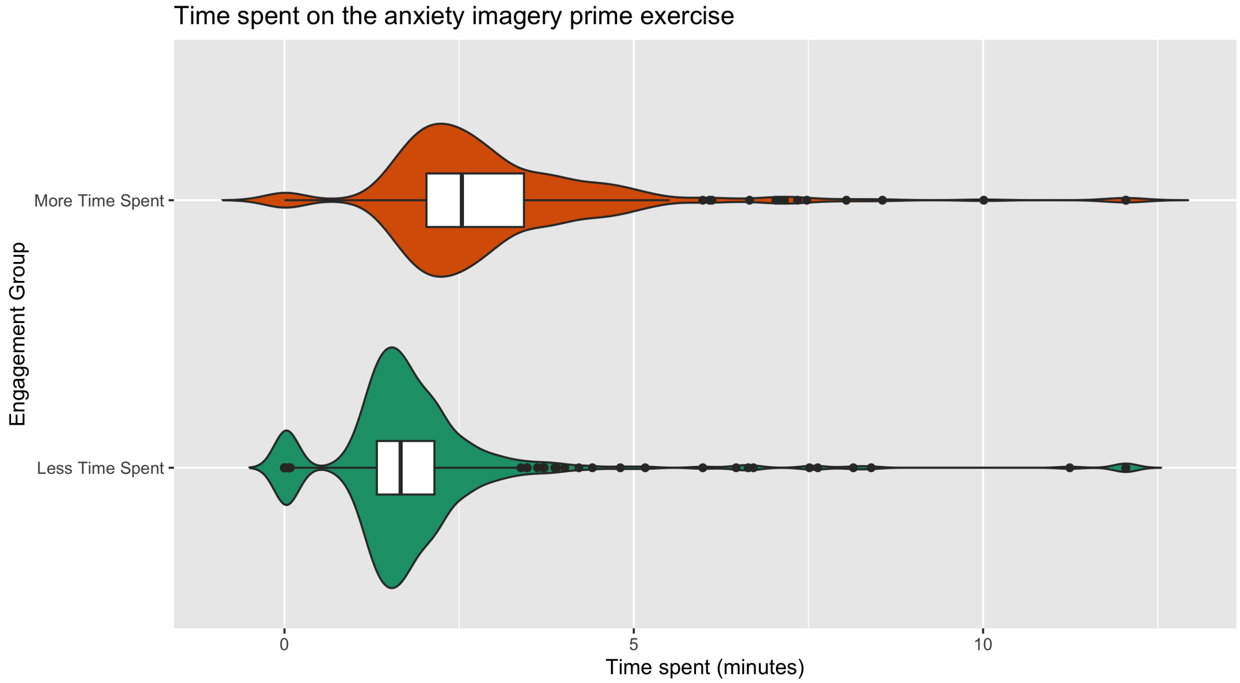

Fig C.1: Box and Violin Plots of Time on Training Components by Engagement Group (Continued)

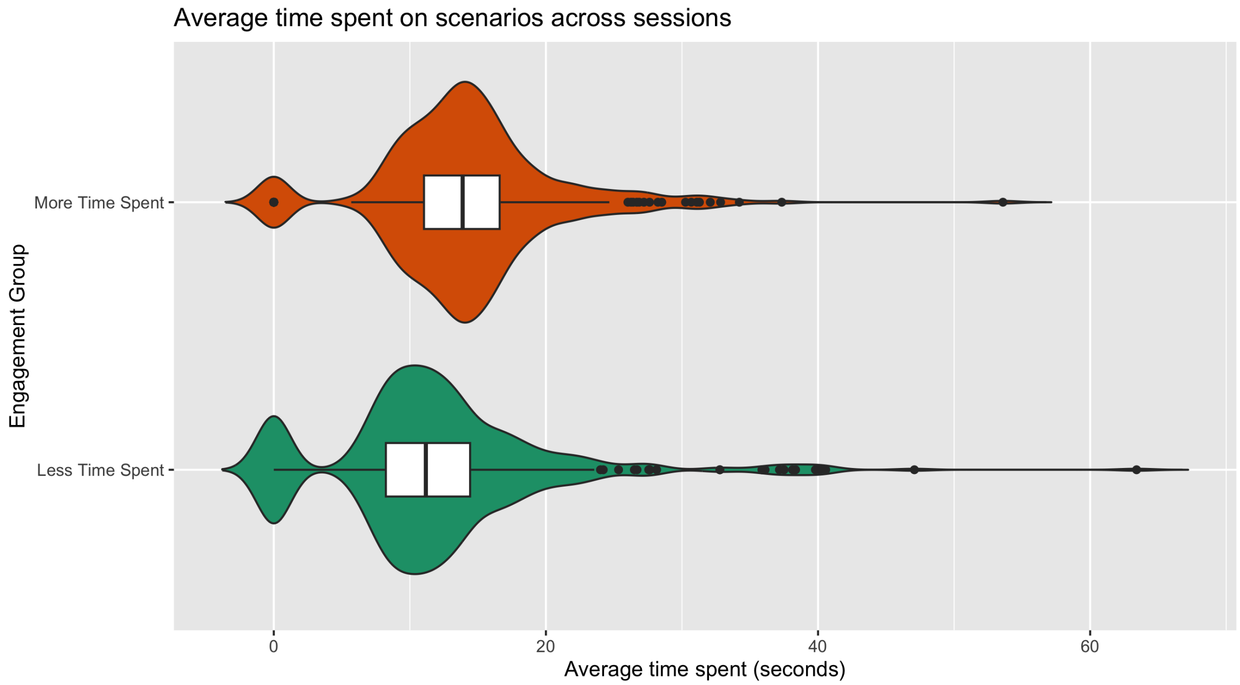

Fig C.2: Box Plots of the Log of Time on Assessment Measures by Engagement Group

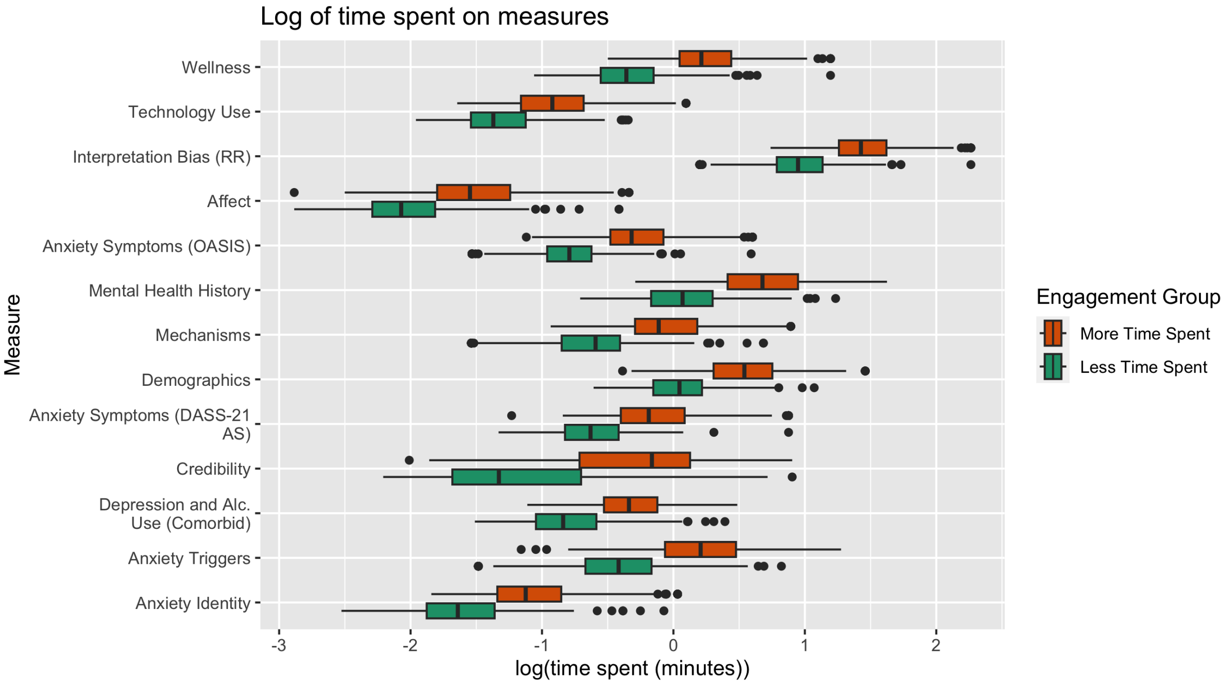

Note. For the repeated measures (Anxiety Symptoms, Anxiety Identity, Interpretation Bias, Depression and Alc. Use (Comorbid), Wellness, Mechanisms, and Affect) the average time spent was used. For more information about these assessment measures see Table 1. RR = Recognition Ratings, OASIS = Overall Anxiety Severity and Impairment Scale; DASS-21 AS = Anxiety Scale of Depression Anxiety Stress Scales.

Fig C.3: Density Distributions of the Log of Time on Assessment Measures by Engagement Group

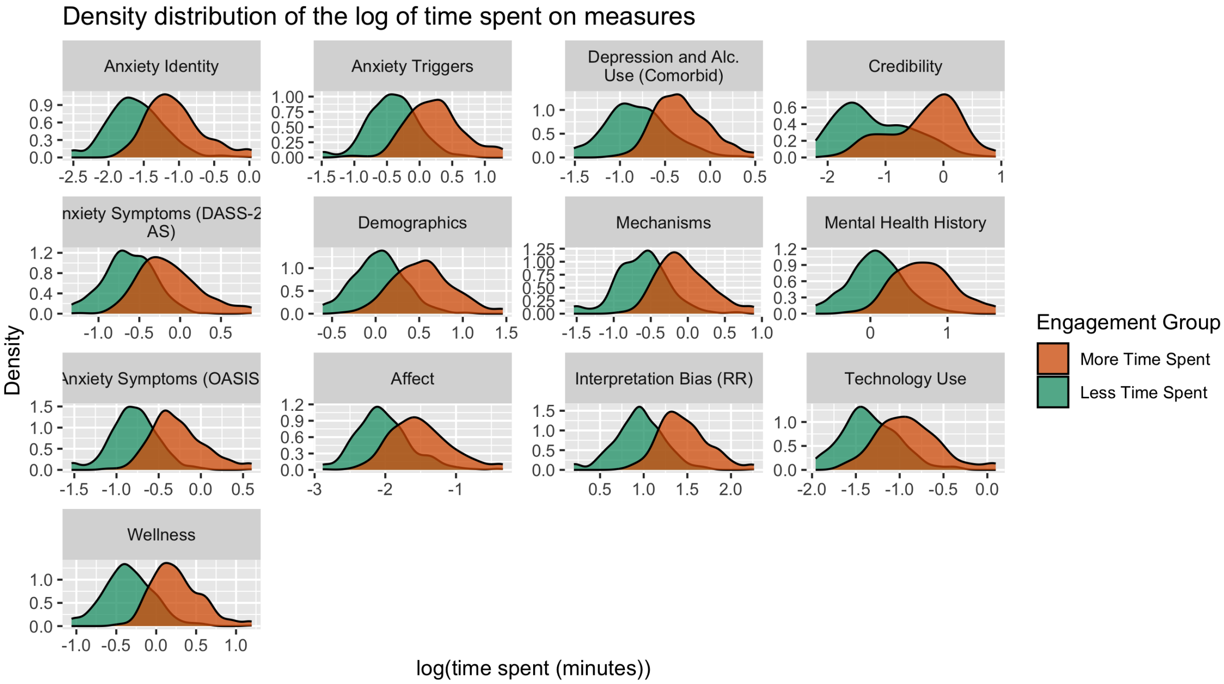

Note. For the repeated measures (Anxiety Symptoms, Anxiety Identity, Interpretation Bias, Depression and Alc. Use (Comorbid), Wellness, Mechanisms, and Affect) the average time spent was used. For more information about these assessment measures see Table 1. RR = Recognition Ratings, OASIS = Overall Anxiety Severity and Impairment Scale; DASS-21 AS = Anxiety Scale of Depression Anxiety Stress Scales.

Fig C.4: Density Distribution of Task Completion Rate by Engagement Group

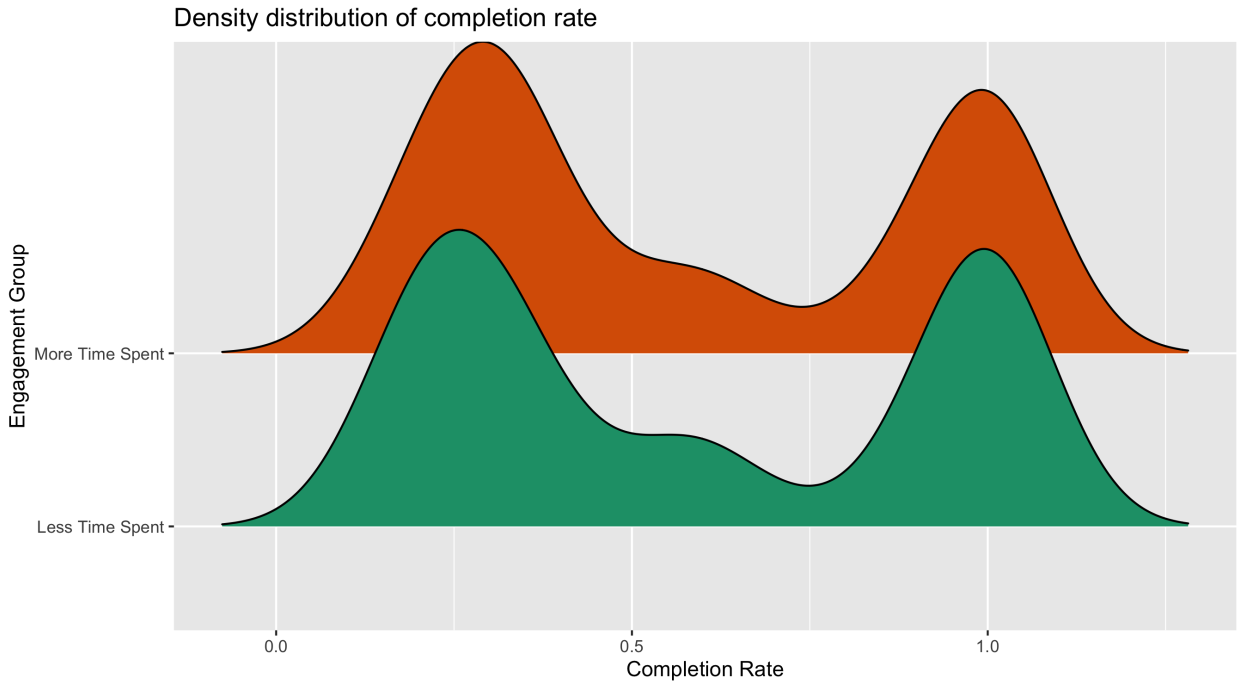

Fig C.5: Model-Estimated Means Over Time by Engagement Group for Sensitivity Analysis

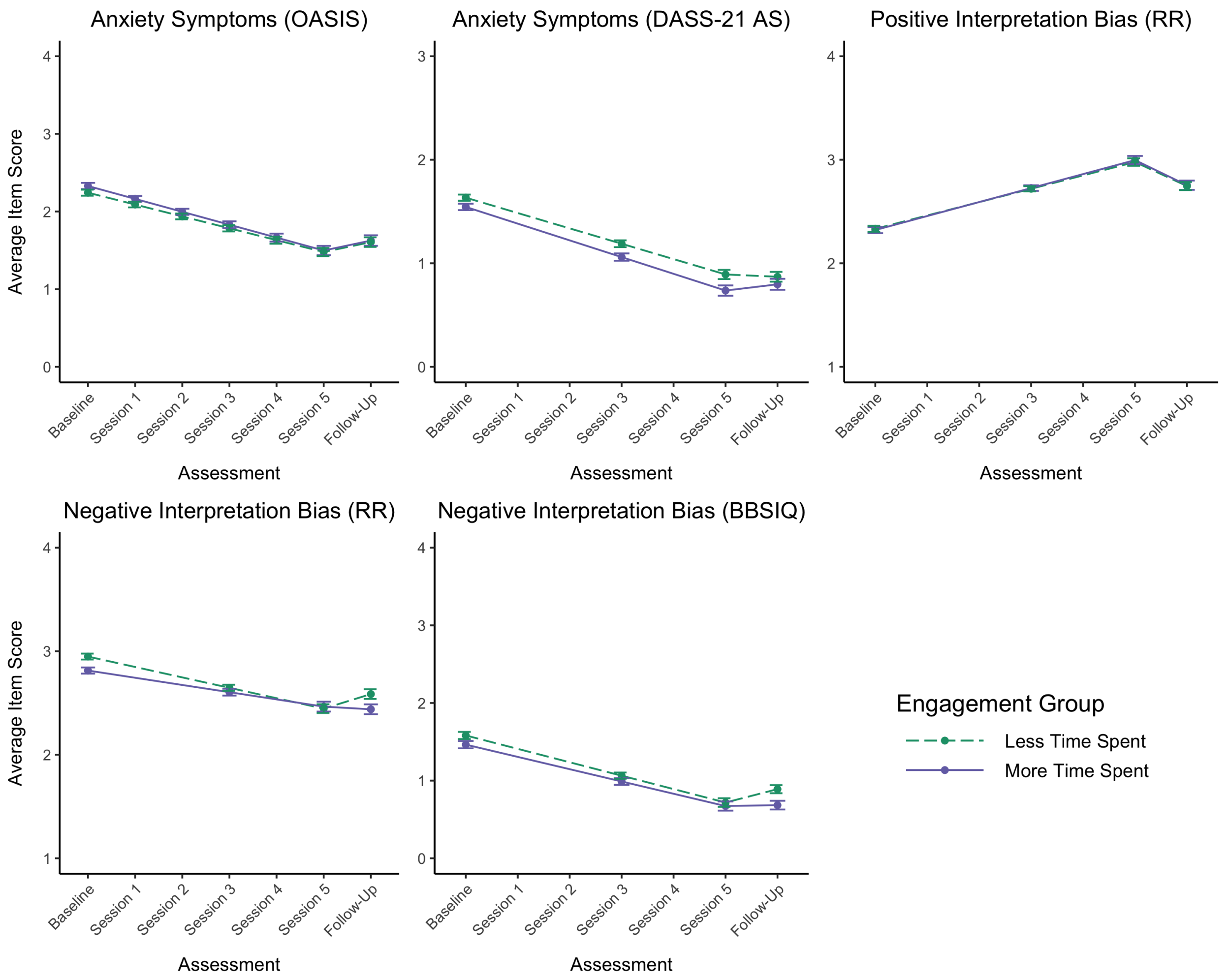

Note. Estimated means (±1 *SE*) from the piecewise linear multilevel models at mean level of training confidence were calculated in each computed dataset and pooled across datasets using the `testConstraints` function of the `mitml` package (ver 0.4-3) [40]. Plots were created with the `ggplot2` (ver. 3.3.5) [41] and `cowplot` (ver. 1.1.1) [42] packages. Estimates are shown only for timepoints at which the measure was assessed. OASIS = Overall Anxiety Severity and Impairment Scale; DASS-21 AS = Anxiety Scale of Depression Anxiety Stress Scales, RR = Recognition Ratings, BBSIQ = Brief Body Sensations Interpretation Questionnaire.

Fig C.6: Visualization of Clusters Using Principal Components for 2, 3, and 4 Clusters

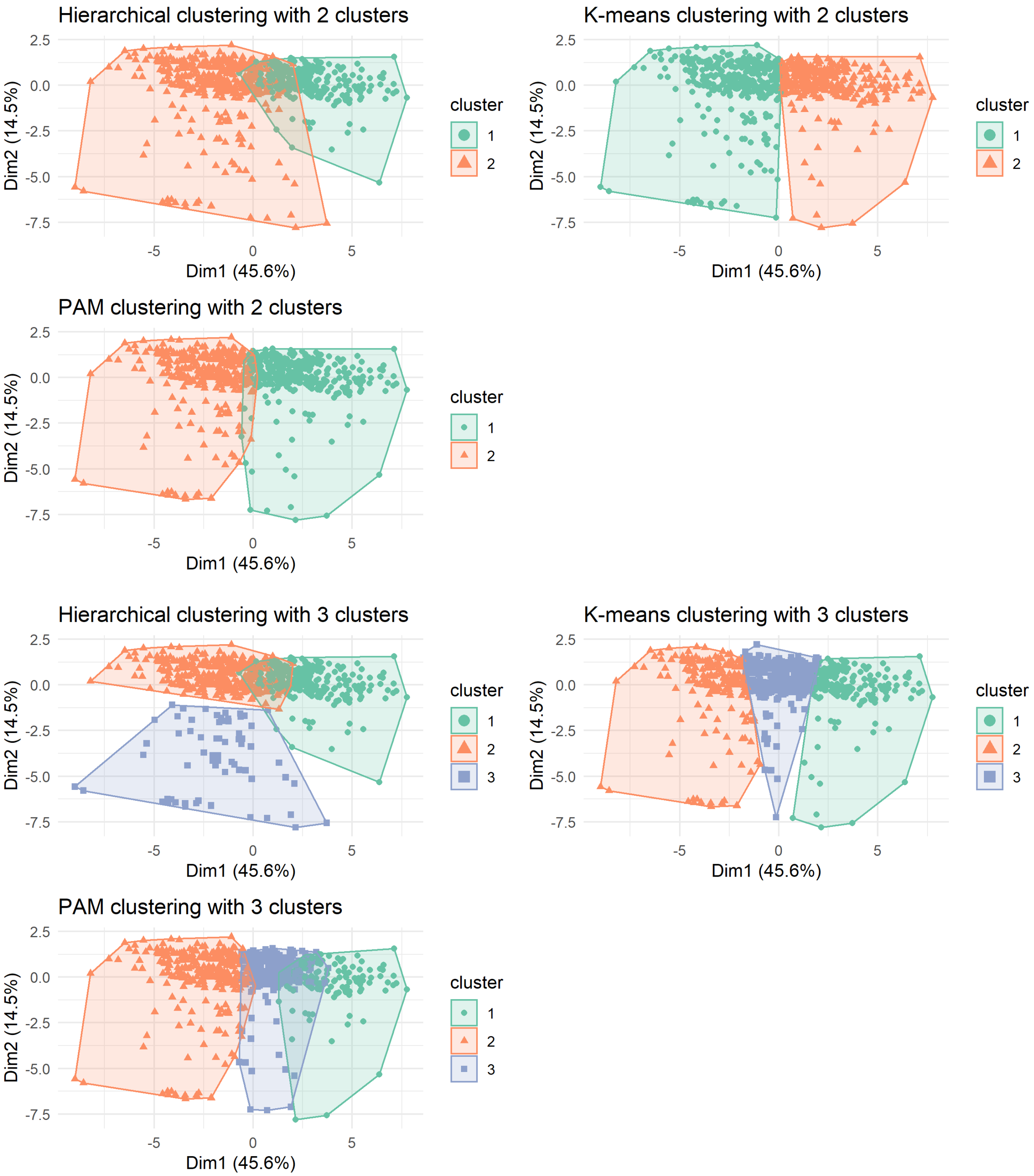

Fig C.6: Visualization of Clusters Using Principal Components for 2, 3, and 4 Clusters (cont.)

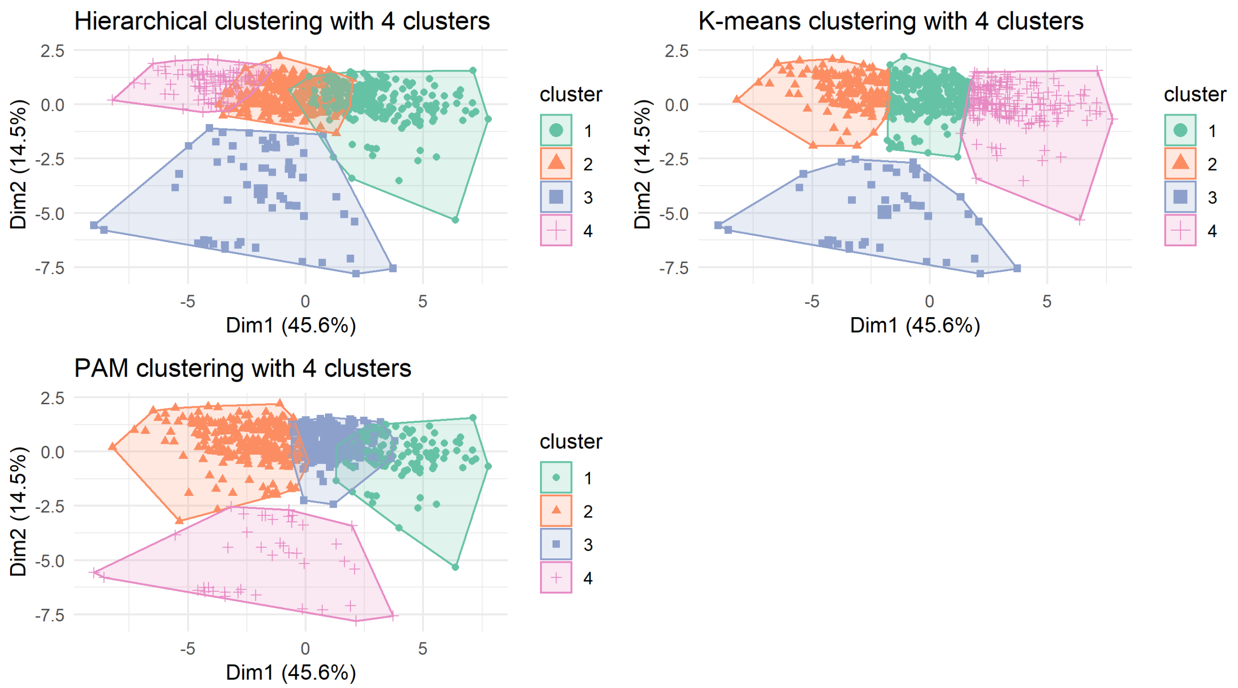

Note. Plots were created with the `fviz_cluster` function of the `factoextra` package (ver. 1.0.7). [21] The axes Dim1 and Dim2 represent the first two principal components from principal components analysis. The percentages shown (45.6% for Dim1, 14.5% for Dim2) represent the amount of variance explained by each principal component. PAM = partitioning around medoids.

References

1. Li Y, Guo Y, Hong YA, Zeng Y, Monroe-Wise A, Zeng C, et al. Dose-Response Effects of Patient Engagement on Health Outcomes in an mHealth Intervention: Secondary Analysis of a Randomized Controlled Trial. JMIR Mhealth Uhealth. 2022;10(1):e25586. doi: 10.2196/25586.

2. James G, Witten D, Hastie T, Tibshirani R. Unsupervised Learning. In: An Introduction to Statistical Learning with Applications in R. 1st ed. New York, NY: Springer; 2013. pp. 373–418. doi: 10.1007/978-1-4614-7138-7.

3. Madhulatha TS. Comparison between K-Means and K-Medoids Clustering Algorithms. In: Wyld DC, Wozniak M, Chaki N, Meghanathan N, Nagamalai D, editors. Advances in Computing and Information Technology. Berlin, Heidelberg: Springer Berlin Heidelberg; 2011. pp. 472–481.

4. Schubert E, Rousseeuw PJ. Faster k-Medoids Clustering: Improving the PAM, CLARA, and CLARANS Algorithms. In: Amato G, Gennaro C, Oria V, Radovanović M, editors. Similarity Search and Applications. SISAP 2019. Lecture Notes in Computer Science, vol 11807. Springer; 2019. pp. 171–187. doi: 10.1007/978-3-030-32047-8_16.

5. Case Western Reserve University. Readiness Ruler; 2021. Available from: `https://case.edu/socialwork/centerforebp/resources/readiness-ruler`.

6. Kroenke K, Spitzer RL, Williams JBW. The Patient Health Questionnaire-2: Validity of a Two-Item Depression Screener. Med Care. 2003;41(11):1284. doi: 10.1097/01.MLR.0000093487.78664.3C.

7. Bush K, Kivlahan DR, McDonell MB, Fihn SD, Bradley KA, for the Ambulatory Care Quality Improvement Project (ACQUIP). The AUDIT Alcohol Consumption Questions (AUDIT-C): An Effective Brief Screening Test for Problem Drinking. Arch Intern Med. 1998;158(16):1789–1795. doi: 10.1001/archinte.158.16.1789.

8. Ersner-Hershfield H, Garton MT, Ballard K, Samanez-Larkin GR, Knutson B. Don't stop thinking about tomorrow: Individual differences in future self-continuity account for saving. Judgm Decis Mak. 2009;4(4):280–286. doi: 10.1017/S1930297500003855.

9. Dennis JP, Vander Wal JS. The Cognitive Flexibility Inventory: Instrument Development and Estimates of Reliability and Validity. Cogn Ther Res. 2010;34(3):241–253. doi: 10.1007/s10608-009-9276-4.

10. Francis AW, Dawson DL, Golijani-Moghaddam N. The development and validation of the Comprehensive assessment of Acceptance and Commitment Therapy processes (CompACT). J Contextual Behav Sci. 2016;5(3):134–145. doi: 10.1016/j.jcbs.2016.05.003.

11. Gross JJ, John OP. Individual differences in two emotion regulation processes: Implications for affect, relationships, and well-being. J Pers Soc Psychol. 2003;85(2):348–362. doi: 10.1037/0022-3514.85.2.348.

12. Carleton RN, Norton MAPJ, Asmundson GJG. Fearing the unknown: A short version of the Intolerance of Uncertainty Scale. J Anxiety Disord. 2007;21(1):105–117. doi: 10.1016/j.janxdis.2006.03.014.

13. Chen G, Gully SM, Eden D. Validation of a New General Self-Efficacy Scale. Organ Res Methods. 2021;4(1):62–83. doi: 10.1177/109442810141004.

14. Dweck CS. Mindset: The new psychology of success. New York, NY: Random House; 2006.

15. Scheier MF, Carver CS, Bridges MW. Distinguishing optimism from neuroticism (and trait anxiety, self-mastery, and self-esteem): A reevaluation of the Life Orientation Test. J Pers Soc Psychol. 1994;67(6):1063–1078. doi: 10.1037/0022-3514.67.6.1063.

16. Cheung F, Lucas RE. Assessing the validity of single-item life satisfaction measures: results from three large samples. Qual Life Res. 2014;23(10):2809–2818. doi: 10.1007/s11136-014-0726-4.

17. Wolpe J. The practice of behavior therapy. Oxford, England: Pergamon; 1969.

18. Leys C, Ley C, Klein O, Bernard P, Licata L. Detecting outliers: Do not use standard deviation around the mean, use absolute deviation around the median. J Exp Soc Psychol. 2013;49(4):764–766. doi: 10.1016/j.jesp.2013.03.013.

19. Guha S, Rastogi R, Shim K. CURE: an efficient clustering algorithm for large databases. ACM SIGMOD Rec. 1998;27(2):73–84. doi: 10.1145/276305.276312.

20. Brock G, Pihur V, Datta S, Datta S. clValid: An R Package for Cluster Validation. J Stat Softw. 2008;25(4):1–22. doi: 10.18637/jss.v025.i04.

21. Kassambara A, Mundt F. factoextra: Extract and Visualize the Results of Multivariate Data Analyses; 2020. Available from: `https://CRAN.R-project.org/package=factoextra`.

22. Charrad M, Ghazzali N, Boiteau V, Niknafs A. NbClust: An R Package for Determining the Relevant Number of Clusters in a Data Set. J Stat Softw. 2014;61(6):1–36.

23. Ezugwu AE, Ikotun AM, Oyelade OO, Abualigah L, Agushaka JO, Eke CI, et al. A comprehensive survey of clustering algorithms: State-of-the-art machine learning applications, taxonomy, challenges, and future research prospects. Eng Appl Artif Intell. 2022;110:104743. doi: 10.1016/j.engappai.2022.104743.

24. Hastie T, Tibshirani R, Friedman J. Unsupervised Learning. In: Hastie T, Tibshirani R, Friedman J, editors. The Elements of Statistical Learning: Data Mining, Inference, and Prediction. New York, NY: Springer; 2009. pp. 485–585. doi: 10.1007/978-0-387-84858-7_14.

25. R Core Team. R: A Language and Environment for Statistical Computing. Vienna, Austria: R Foundation for Statistical Computing; 2023. Available from: `https://www.R-project.org/`.

26. Duda RO, Hart PE, Stork DG. Pattern Classification. 2nd ed. Wiley; 2000.

27. Hennig C. fpc: Flexible Procedures for Clustering; 2024. Available from: `https://cran.r-project.org/web/packages/fpc/index.html`.

28. Toffalini E, Girardi P, Giofrè D, Altoè G. Entia Non Sunt Multiplicanda … Shall I look for clusters in my cognitive data? PLoS One. 2022;17(6):e0269584. doi: 10.1371/journal.pone.0269584.

29. Huang H, Liu Y, Hayes DN, Nobel A, Marron JS, Hennig C. Significance Testing in Clustering. In: Hennig C, Meila M, Murtagh F, Rocci R, editors. Handbook of Cluster Analysis. Chapman and Hall/CRC; 2015. pp. 315–333.

30. Steinley D, Brusco MJ. Choosing the number of clusters in K-means clustering. Psychol Methods. 2011;16(3):285–297. doi: 10.1037/a0023346.

31. Enders CK, Du H, Keller BT. A model-based imputation procedure for multilevel regression models with random coefficients, interaction effects, and nonlinear terms. Psychol Methods. 2020;25(1):88–112. doi: 10.1037/met0000228.

32. Enders CK, Keller BT, Du H, Levy R. Blimp Studio; 2022. Available from: `https://www.appliedmissingdata.com/blimp`.

33. Eberle JW, Daniel KE, Baee S, Silverman AL, Lewis E, Baglione AN, et al. Web-based interpretation bias training to reduce anxiety: A sequential, multiple-assignment randomized trial. J Consult Clin Psychol. 2024;92(6):367–384. doi: 10.1037/ccp0000896.

34. Grund S, Lüdtke O, Robitzsch A. Multiple Imputation of Missing Data for Multilevel Models: Simulations and Recommendations. Organ Res Methods. 2018;21(1):111–149. doi: 10.1177/1094428117703686.

35. Keller BT, Enders CK. Blimp user's guide (Version 3); 2021. Available from: `www.appliedmissingdata.com/multilevel-imputation.html`.

36. Enders CK. Applied missing data analysis. New York: Guilford Press; 2010. p. 265.

37. Wyatt KP, Eberle JW, Ruork AK, Neacsiu AD. Mechanisms of change in treatments for transdiagnostic emotion dysregulation: The roles of skills use, perceived control, and mindfulness. Clin Psychol Psychother. 2023;30(6):1380–1392. doi: 10.1002/cpp.2879.

38. Brown VA. An Introduction to Linear Mixed-Effects Modeling in R. Adv Methods Pract Psychol Sci. 2021;4(1):251524592096035. doi: 10.1177/2515245920960351.

39. Cohen J. Statistical Power Analysis for the Behavioral Sciences. 2nd ed. New York: Routledge; 1988.

40. Grund S, Robitzsch A, Luedtke O. mitml: Tools for Multiple Imputation in Multilevel Modeling; 2021. Available from: `https://CRAN.R-project.org/package=mitml`.

41. Wickham H, Chang W, Henry L, Pedersen TL, Takahashi K, Wilke C, et al.. ggplot2: Create Elegant Data Visualisations Using the Grammar of Graphics; 2022. Available from: https://CRAN.R-project.org/package=ggplot2.

42. Wilke CO. cowplot: Streamlined Plot Theme and Plot Annotations for 'ggplot2'; 2020. Available from: https://CRAN.R-project.org/package=cowplot.
