# Supplementary material for: Behavioral engagement patterns and psychosocial outcomes in web-based interpretation bias training for anxiety
Source: PLOS Digit Health. 2025 Jul 24;4(7):e0000945. doi: 10.1371/journal.pdig.0000945 (PMC12289040; doi:10.1371/journal.pdig.0000945)
Supplement: Attachment — Submitted filename: Response to Reviewers From Prior Submission.pdf [file pdig.0000945.s002.pdf]

Response to Reviewers' Comments to the Authors

Thank you very much again for the opportunity to address Reviewer 4's remaining concerns, which we address point by point below.

Reviewer 3

While I am not a quantitative researcher, I can assess the clarity and soundness of the argument. You have made great efforts to address my concerns and provided some interesting reasons how time is compromised by cognition and literacy. I think this paper makes the case that time is not a good marker of participant engagement for many reasons.

Response: We thank the reviewer for their kind words! We appreciate their feedback and their note in the prior round of review that all of their comments have been addressed.

Reviewer 4

I appreciate the great efforts made by the authors to make revisions and positive response to reviewers' comments, with a large amount of supplementary materials clearly listing out the process of data analysis. I don't doubt about the rigor and robustness of these statistical analyses per se (cluster analysis/ multi-level regression). However, there are still major concerns to be addressed about the rationale, interpretation and implication underlying these sophisticated and massive analyses and results.

Response: We appreciate the reviewer's recognition of the clarity, rigor, and sophistication of our analyses and are grateful for the opportunity to further clarify the rationale for our analyses and the interpretation and implications of our results. As we note below, we believe that some of the concerns the reviewer raised may have stemmed from their taking a different perspective on a few points in our paper. We hope that our revisions help clarify our perspective on these points.

Point 1

First, it's confusing why authors include time on assessment tasks in the engagement marker. When people talk about the intensity/ dosage of interventions, they usually only refer to the active and effective components of intervention (should only be the training components in this CBM-I). In contrast, the assessment tasks are usually deemed as an "independent" process apart from the interventions. It is worrying that the inclusion of assessment tasks in engagement marker will:
(1) make the methodology and results incomparable to previous studies;
(2) blur the definition and interpretation of task completion rate (not traditionally what people concern about, i.e., completion rate of training session);
(3) blur the definition and interpretation of overall engagement marker, since most of time in this marker is actually composed of the tedious assessment tasks (based on Table 2, training time is ~10-13 min while assessment time is ~ 9-16 minutes, taking up 50% of overall time). In another word, if we change different assessment scales (let say make it shorter and easier to

complete), then this engagement marker may have significant and systematic change in its construction.

Response: We appreciate this prompt to further clarify our rationale for defining engagement markers (i.e., task completion rate and time spent metrics) based on assessment tasks in addition to training tasks. We included assessment tasks in our engagement markers for a few reasons.

First, some prior studies have also considered assessment tasks as relevant to engagement. For example, in CBM-I participants from the same parent trial (Eberle et al., 2024), our team used time-related (and other) features from both assessment and training tasks in a machine learning algorithm that predicted dropout with high accuracy (F_1 = .92; Baee et al., 2024). Studies from other teams have also included assessment-like tasks in engagement variables that are then used to predict treatment outcomes. For instance, time spent on measures of symptoms, daily activities, mood, processes of change, and self-efficacy were included in total website usage variables that were then used to predict outcomes in web-based programs for depression (Clarke et al., 2009) and physical activity (Wanner et al., 2009). Moreover, a scoping review found that assessment task features (e.g., number of measures completed or frequency of completing measures of treatment targets, symptoms, and outcomes) are often used as markers of engagement in apps for mental and physical health (Pham et al., 2019). However, to be clear that not all studies include assessment-related engagement markers, we now note in the Limitations section (p. 26) that “not all studies include assessment-related markers.”

Second, although we agree that the training tasks are likely the most active components of the program, we believe the assessment tasks are also important for several reasons. One reason is that monitoring treatment outcomes improves efficacy and reduces dropout (de Jong et al., 2021), and participants were informed early on that they would receive feedback about their changes in anxiety symptoms (assessed via the OASIS) at the end of the study. Moreover, assessment itself (even without feedback) can produce clinically desirable reactive effects that improve efficacy (Korotitsch & Nelson-Gray, 1999). In addition, at each session the training and assessment tasks are presented sequentially and seamlessly through the same interface; one assessment task (i.e., Affect) appears before the training tasks, and the training tasks are followed immediately by the remaining assessment tasks, some of which have a format very similar to the training tasks. For example, the Recognition Ratings (RR) measure of interpretation bias, like the CBM-I scenarios, consists of a series of emotionally ambiguous scenarios ending in a word fragment followed by a comprehension question (but unlike CBM-I scenarios, neither the word fragment nor the question resolves the ambiguity). The Brief Body Sensations Interpretation Questionnaire (BBSIQ) also involves a series of emotionally ambiguous scenarios. Thus, although in the paper we have used the terms *training tasks* and *assessment tasks* to provide an organizational framework for the reader, this distinction is likely not as clear from the user’s perspective. Furthermore, as noted above, in the same parent trial (Eberle et al., 2024), self-reported responses (i.e., demographics, state anxiety) and passive features (e.g., time spent) from baseline and Session 1 assessment tasks were used in a machine learning algorithm to classify which users were at high risk of dropping out, which in turn determined whether users would be randomized to supplemental telecoaching. Such tailoring variables are key for making treatment decisions in adaptive interventions (e.g., using baseline assessment data to decide which initial treatment to offer a user or using later data to decide which users are not responding and how to

adjust treatment for nonresponders; Lei et al., 2012). For these reasons, we did not view assessment tasks as fully independent from training tasks, and thus allowed all tasks to inform the clusters' potentially complex engagement patterns.

Third, including engagement markers based on assessment tasks allowed us to focus our analysis on the intent-to-treat (ITT) sample of CBM-I participants (i.e., the full set of participants who had been randomly assigned to CBM-I, before any such users dropped out). We noted our focus on this sample on page 6: "We focused on participants who started the first of five weekly training sessions." The ITT sample is important because it enables tests of the causal effects of randomly assigned treatment conditions. Although we do not compare CBM-I participants to participants randomized to other conditions in the present analyses, focusing on the ITT sample allows our results to generalize to other ITT samples of CBM-I participants (the typical sample of interest). Focusing on this sample also allowed us to analyze engagement from the start of the program, when engagement is highly variable and thus key to understand. Including engagement markers based on assessment tasks allowed us to analyze the ITT sample because if we had included a smaller number of time-related markers (e.g., markers based on only training tasks), we likely would have obtained clusters that we would have been unable to compare on treatment outcomes unless we restricted the ITT sample further. Specifically, we likely would have found two clusters with low versus high task completion rates and likely would have had insufficient outcome data for the low completion rate group.[1] The low completion rate group would have less outcome data than the high completion rate group because the training and assessment tasks were administered in series (vs. parallel), meaning that if participants did not complete a training or assessment task they were unable to complete any subsequent training and assessment tasks. (We already noted a related limitation on p. 26 that prevented "analysis of completion of training tasks separate from assessment tasks.") To avoid lacking sufficient outcome data for one cluster, in this situation we would have to restrict the sample to users who had progressed to a certain later point in the study, losing our focus on the ITT sample. However, including time-related engagement markers based on the full set of training and assessment tasks (which also provided consistency with our task completion rate marker based on the full set of training and assessment tasks) had the fortunate byproduct of yielding clusters with sufficient outcome data for comparison in the ITT sample.

In summary, including engagement markers based on assessment tasks (a) has some precedence in prior studies, (b) allowed the assessment tasks (which we viewed as theoretically relevant and not fully independent from the training tasks) to inform users' complex engagement patterns, and (c) had the fortuitous outcome of allowing us to focus on the ITT sample (the typical sample of interest and one that permitted analysis of engagement from the start of the program).

[1] This is suggested by the bimodal distribution of task completion rate (Figure S3.4), and, indeed, we found two low vs. high completion rate groups when we included a smaller number of time-related training and assessment markers in our initial cluster analysis. However, as we noted in Footnote 2 of the manuscript (p. 9), because most participants in the low completion rate group of our initial cluster analysis did not finish the first training session and thus had assessment data only at baseline, the imputation model used to handle missing outcome data at later time points did not converge. This would have prevented appropriate missing data handling in the multilevel models used to analyze differences between engagement groups in treatment outcomes, or required us to restrict the sample to participants with more assessment data, in turn losing our desired focus on the full ITT sample.

To clarify our rationale for including assessment tasks in the paper, we have expanded the first paragraph of the Engagement Markers section of the Method (p. 9), which now reads:

> We defined program use markers for a cluster analysis (see below) based on task completion rate and time spent on training and assessment tasks.[2] Some prior studies have also treated assessment tasks as relevant to engagement, using rates of completing assessment tasks as engagement markers (Pham et al., 2019) or including time spent on assessment tasks in predictors of dropout (Baee et al., 2024) or treatment outcomes (Clarke et al., 2009; Wanner et al., 2009). We viewed assessment tasks as relevant to engagement in the program for a few reasons. First, interventions can include assessments of their targets and outcomes solely to improve efficacy by providing progress feedback (de Jong et al., 2021; which users were told they would receive for their OASIS scores at the end of the parent trial) or by eliciting desirable reactive effects from the assessment task itself, even without feedback (Korotitsch & Nelson-Gray, 1999). Second, the training and assessment tasks, some of which had similar formats (e.g., the CBM-I training task and RR and BBSIQ assessment tasks all present ambiguous scenarios), were slightly intermixed and presented seamlessly at training sessions; thus, users may not have viewed all training and assessment tasks as distinct. Third, interventions can include assessments to adapt treatment based on certain tailoring variables (Lei et al., 2012), as the parent trial used self-reported responses (i.e., demographics, state anxiety) and passive features (e.g., time spent) from assessment tasks to classify users at risk of dropout (Eberle et al., 2024). Given that we did not view training and assessment tasks as fully independent, we allowed all tasks to inform the clusters' potentially complex engagement patterns.

We also now further clarify that our clusters reflect patterns of engagement in the entire program (including both training and assessment tasks) by providing several reminders. Specifically, in the Abstract (p. 2), Objective (p. 5), Results (pp. 16, 18), Discussion (pp. 23-24), and Conclusion (p. 27), we now refer to "participants who generally spent less time on the program (including both training and assessment tasks)…" rather than only "participants who spent less time…" These reminders supplement our statements throughout that we "extracted program use markers based on task completion rate and time spent on training and assessment tasks" (e.g., p. 2).

Finally, we would also like to note that it is not quite true that assessment tasks were responsible for 50% of users' time spent on tasks. Rather, over the course of the study, the Less Time Spent group spent 48.8% more time on training tasks than on assessment tasks (39.81 min and 26.75 min, respectively), and the More Time Spent group spent 12.9% more time on training tasks than on assessment tasks (50.25 min and 44.51 min, respectively).

New references added to paper:

Clarke, G., Kelleher, C., Hornbrook, M., DeBar, L., Dickerson, J., & Gullion, C. (2009). Randomized effectiveness trial of an Internet, pure self-help, cognitive behavioral intervention for depressive symptoms in young adults. *Cognitive Behaviour Therapy, 38*(4), 222-234. https://doi.org/10.1080/16506070802675353

de Jong, K., Conijn, J. M., Gallagher, R. A., Reshetnikova, A. S., Heij, M., & Lutz, M. C. (2021). Using progress feedback to improve outcomes and reduce drop-out, treatment duration, and deterioration: A multilevel meta-analysis. *Clinical Psychology Review, 85*. https://doi.org/10.1016/j.cpr.2021.102002

Korotitsch, W. J., & Nelson-Gray, R. O. (1999). An overview of self-monitoring research in assessment and treatment. *Psychological Assessment, 11*(4), 415. https://psycnet.apa.org/doi/10.1037/1040-3590.11.4.415

Lei, H., Nahum-Shani, I., Lynch, K., Oslin, D., & Murphy, S. A. (2012). A" SMART" design for building individualized treatment sequences. *Annual Review of Clinical Psychology, 8*(1), 21-48. https://doi.org/10.1146/annurev-clinpsy-032511-143152

Wanner, M., Martin-Diener, E., Braun-Fahrländer, C., Bauer, G., & Martin, B. W. (2009). Effectiveness of active-online, an individually tailored physical activity intervention, in a real-life setting: Randomized controlled trial. *Journal of Medical Internet Research, 11*(3). http://dx.doi.org/10.2196/jmir.1179

Point 2

Second, I agree with the comment from one reviewer that the necessity and rationale of using cluster analysis. Given one-time, linear and consecutive tasks in CBM-I, it's very easy and reasonable to just sum up the overall use time. Cluster analysis is normally used to cluster groups based on multiple variables that have very different attributes (e.g., different demographic variables, different outcome performances). However, in this study, most variables (except for the task completion rate) put into cluster analysis have similar attributes (time) and are highly related to each other. In general, I would say using cluster analysis here has more harm (vague interpretation and implication) than good. Instead, it seems better to use three component outcomes independently (task completion rate, training time, assessment time).

Response: We thank the reviewer for their feedback, which has led us to further clarify the rationale for the cluster analysis. In particular, we have further clarified the distinction we had tried to explain between “variable-centered” and “person-centered” approaches to research in the Background and Cluster Analysis section of the Results. Variable-centered research focuses on the unique, average effects of one or a few variables (e.g., behavioral engagement) on treatment outcomes. In terms of the three variables the reviewer proposed, a variable-centered approach might ask the question “What is the effect of task completion rate on change in anxiety?” and analyze this question with a multilevel model that regresses anxiety on task completion rate, time (i.e., anxiety assessment point), and the interaction between task completion rate and time (with a focus on the interaction). The same model could be used to analyze the effects of training time and assessment time, using a separate model for each of these two predictors, where training time and assessment time are the mean time spent across training tasks and the mean time spent across assessment tasks, respectively. (Alternatively, all three predictors–task completion rate, training time, assessment time–could be put in one model to estimate each predictor’s unique effect.) This approach might yield findings such as “on average across participants, greater time spent on training tasks is associated with greater change in anxiety [after controlling for task completion rate and time spent on assessment tasks, if these predictors are included in the same model].”

Although these are interesting variable-centered research questions, our research question was different in that it originated from a person-centered approach to research. In a person-centered approach, the focus is on differences in treatment outcomes between clusters of participants who are defined by different complex configurations of values across a set of variables. That is, rather than aggregating *P* engagement variables by type to create a few 1-dimensional predictors and analyzing the (unique) effect of each aggregated predictor on treatment outcomes, the focus in person-centered research starts by (a) imagining a complex, multidimensional (*P*-dimensional) space in which each participant has a data vector representing that participant's unique location along all of the *P* dimensions (based on all the participant's values on the *P* variables). It goes on to (b) consider how different participants' locations in this multidimensional space relate to one another, and the extent to which some participants' locations are closer to one another than to the locations of other participants, starting to form clusters of participants, where each cluster reflects a unique configuration, pattern, or set of values across all of the *P* variables. Finally, the approach (c) analyzes whether these different *P*-dimensional clusters have different treatment outcomes. In this way, the person-centered approach asks, "How do different data patterns in a complex, high dimensional space of individual engagement variables relate to treatment outcomes?"

We took a person-centered approach to analyze engagement as a more complex phenomenon than is possible from a variable-centered approach. Prior variable-centered research that has modeled engagement as a 1-dimensional predictor has yielded mixed results on the average relation between engagement and treatment outcomes (e.g., Donkin et al., 2011), highlighting the need to consider more nuanced patterns, given that engagement is a multifaceted construct. Cluster analysis is an appropriate and standard method for person-centered research (Bauer & Shanahan, 2007; Howard & Hoffman, 2017) because, as we stated in the Background (p. 5), its "use of multiple (vs. single or composite) metrics and its exploratory nature can suggest clusters that differ based on potentially more complex engagement patterns in the data than can be represented by single metrics or predicted a priori."

As described by Howard and Hoffman (2018; pp. 3-4), "The person-centered approach (e.g., mixture models, cluster analyses) is quickly growing in popularity due to the finer-grained detail that it allows…This approach is used to identify the dynamics of emergent subpopulations in a sample based on a set of chosen variables, and it is appropriate for investigating research questions and hypotheses aimed at (a) categorizing subjects into common subpopulations based on substantive variables and (b) understanding the relations of these subpopulations with predictors, correlates, or outcomes…Subpopulations may be based on very complex patterns of many variables." Moreover, Bauer and Shanahan (2007, p. 256) state that person-centered analyses "identify key patterns of values across variables, where the person, viewed holistically, is the unit of analysis" and that person-centered "methods that identify configurations of variables–quite typically, cluster analyses…–are capable of appreciating the high level of interaction and nonlinear relationships…" of a system and "result in the identification of a small set of clusters or classes that then supplant the individual variables as predictors or outcomes in subsequent data analysis."

By choosing to use cluster analysis for this paper, we sought to allow each of 17 engagement markers to define participants' engagement patterns, using all available information to define the

patterns in an exploratory way, rather than assuming that markers of a certain type (e.g., involving training vs. assessment tasks) should be collapsed (which would also lose unique information from individual markers). By permitting each marker to contribute meaningful information to define a 17-dimensional configuration of values for each participant that can then be compared to the configurations of other participants, cluster analysis mapped directly onto our research question, whereas other, variable-centered approaches address different kinds of research questions. As we stated in the Background (p. 5), this type of cluster analysis "may be especially useful for studying real-world engagement in implementation settings, where users' varied expectations, goals, needs, and resources may yield more complex engagement patterns than those in efficacy trials.[19]"

Thus, the reviewer's suggestion to collapse our 17 engagement markers into task completion rate, training time, and assessment time and then to regress treatment outcomes on these aggregate predictors would address a variable-centered research question, different from the person-centered research question we sought to address. Regarding the reviewer's comment that our engagement markers are highly related, we excluded markers that had correlations with other markers greater than .70 (this is noted in the Engagement Markers section; p. 11). After excluding such markers, the mean correlation among time spent markers for training tasks was .32 (i.e., an R^2 of .10, or 10.0% of the variance for one marker in a pair), and the mean correlation among time spent markers for assessment tasks was .48 (or 23.2% of the variance for one marker in a pair; see Table S2.13 for all correlations). Although this suggests that neither set of markers has redundant elements, we do note as a limitation (p. 26) that "the retained features still had non-negligible intercorrelations (rs > .1; see Table S2.13) that could have contributed to the algorithm's suggesting two clusters with higher versus lower means across most features (i.e., our More Time Spent and Less Time Spent groups, respectively).[36]"

To further clarify the rationale for cluster analysis in the paper, we now emphasize that analyses of engagement using 1-dimensional predictors have yielded mixed results, highlighting the need to consider more complex engagement patterns. In the Background (p. 4), we have revised the following sentence, which now reads: "Greater use on a given metric is typically expected to yield better outcomes,[23] but mixed findings and methodological differences across studies[24] highlight the need for further research analyzing engagement as a more complex construct." In addition, to better establish the rationale for our cluster analysis (a person-centered method), we have carefully reviewed our paper and made numerous revisions to help clarify that our research question in this paper is person centered and to emphasize that we are studying differences in outcomes for different complex engagement patterns, rather than the average relation between 1-dimensional engagement predictors and outcomes.

Again, we appreciate the reviewer's comment on needing to more fully build the rationale for the cluster analysis.

Point 3

Third, interpretation of results is fuzzy due to use of cluster analysis in this study. Especially, significant demographic differences are detected between more-time vs less-time groups, which can explain differences in use time (pointed out by authors: younger and higher educated patients

have better cognitive, comprehensive and learning ability and therefore use less time in training and assessment; also pointed out by other studies, younger people are more vulnerable to stress and anxiety). Simply applying the two-cluster engagement marker in multi-level regression, without controlling other baseline demographics (also unable to control), will confound the findings between behavioural engagement and psychosocial outcomes. This will also hamper the interpretation and implication of results in the discussion part. In this case, I do not see advantages of cluster analysis over traditional ANCOVA/regression analysis in investigating the "independent" effects of intervention engagement on patient outcomes.

Response: We thank the reviewer for this comment, which allows us to further expand upon our person-centered research question and perspective on these results. As explained in our response to Point 2 above, our person-centered approach did not seek to isolate the unique, average effects of a given engagement variable (e.g., time spent) on treatment outcomes, but rather to determine whether clusters reflecting different complex configurations of values across a set of engagement variables have different outcomes. To the reviewer's point, a variable-centered approach seeking to isolate the unique, independent, unconfounded relation between an engagement variable and outcomes would absolutely face challenges if uncontrolled "third variables" (e.g., demographic or clinical characteristics) confounded the unique effect of the engagement variable on outcomes. However, in a person-centered approach, demographic, clinical, and other variables that did not enter into the cluster analysis provide a means of better understanding the clusters, which (by virtue of being partitions of a multidimensional space) are indeed complex. The reason we ran post hoc tests of cluster differences on demographic and clinical characteristics was not to rule out potential confounds of an engagement-outcome relationship, but rather to better understand the clusters, recognizing patterns of engagement as multifaceted and complex. Thus, although it would technically be possible to control for such characteristics (e.g., age, education) by entering them as covariates in our multilevel models testing the effect of engagement group on treatment outcomes, doing so would defeat the purpose of better understanding the engagement groups, as this would remove relevant information about the actual composition of the two groups. From a person-centered perspective, such demographic and clinical differences are inherent to the actual clusters and relevant to understanding their patterns, not nuisances to be statistically controlled.

As we stated in the Background (p. 4), "examining correlates of use, such as symptom severity and demographic characteristics, may help reveal the meaning of users' behavioral engagement (e.g., lack of time spent on a program likely has a different meaning when a user is higher vs. lower in symptom severity), providing a fuller picture of engagement patterns." Hence, in the Further Characterizing Engagement Groups section of the Discussion (p. 25), we discussed how the clusters' differences in age and education may inform interpretation of their engagement patterns (i.e., it may be "that people in the group that generally spent more time, who were significantly older (medium effect) and less educated (very small effect) than people in the group that spent less time, had slower processing speed[69] and lower reading comprehension.[70]"). We then discussed how such an interpretation of the engagement patterns may help explain our results (i.e., "These differences would likely add to their time (on both training and assessment tasks, as was observed) and potentially make training scenarios less relatable, which could have made it more difficult for participants who spent more time to improve during the training phase. However, once participants who spent more time had improved, perhaps their gains were maintained at follow-up because of their greater physical investment of time during the training

phase.") In this way, the age and education differences provide a fuller picture of the engagement patterns.

We also appreciate the reviewer's interesting comment that other studies show that younger people are more vulnerable to stress and anxiety. Systematically examining the ways that age–and the many cognitive and physical changes age is a proxy for–are associated with both different aspects of engagement and with vulnerability to anxiety will be valuable in future research. However, given that we observed an engagement group difference in anxiety symptoms at baseline on only one anxiety measure (DASS-21 AS but not OASIS), that the difference was very small, and that we did not directly test the relation between age and anxiety, we are reluctant to overinterpret this difference in our data prior to seeing it replicated.

To reiterate our person-centered approach and clarify how such group differences are interpreted in this approach, we have added the following text to the end of the first sentence of the Further Characterizing Engagement Groups section of the Discussion (p. 25).

> Post hoc tests revealed that the groups significantly differed on aspects other than time spent, providing potential insight into our findings for negative interpretation bias. Notably, in our person-centered approach, any engagement group differences on variables that did not enter into the cluster analysis can help inform interpretation of the groups' engagement patterns. (This contrasts with a variable-centered approach, in which any correlates of a given engagement variable would represent potential confounds of the effect of the engagement variable on outcomes.)

Finally, to characterize the engagement groups more fully, we now note the group differences in age and education in the following sentences in the Abstract (p. 2) and first paragraph of the Discussion (p. 23).

> Abstract: "Further, participants who generally spent less time on the program (including both training and assessment tasks) improved in negative interpretation bias (on one of two measures) significantly more during the training phase than those who spent more time (and post hoc tests found were significantly older and slightly less educated)."

> Discussion: "Further, although both groups significantly improved in negative interpretation bias during the training phase, participants who generally spent less time improved in negative bias (when assessed by RR) significantly more than participants who spent more time (and post hoc tests found were significantly older and slightly less educated)."

Point 4

Finally, although there are many significant results in analysis, which is not surprising given the large sample size (n~700) and worthy further discussion, it's unclear that how these statistical differences are meaningful in clinical/ practical aspects. For example, the time differences between high- and low-engagement group are only <1 minutes per excise or <3 second per scenario, and it's hard to judge whether such minor differences are due to the cognitive ability of participants (e.g., age, education) or the willingness/effort to engage. Especially, the training and

tasks in CBM-I are set one-time, fixed, and unrepeatable. I believe that the "behavioural engagement" measured in this study is different from those from previous study (intensity/dosage), actually reflecting the time to complete the training tasks (like a cognitive assessment).

Response: We appreciate the chance to expand on the magnitude of the differences between the groups, the meaning of these differences in behavioral engagement, and our study's potential implications.

Magnitude of Significant Group Differences

In terms of group differences on the engagement markers used to create the groups and on the demographic and clinical variables we used to further characterize the groups post hoc, as stated in the *K*-Means Clustering section of the Method (p. 13), we had computed standardized effect sizes for these differences (*r* for continuous variables; Cramér's *V* for categorical variables), which we interpreted per Cohen's (1988) guidelines. As reported in the Cluster Analysis section of the Results (pp. 15-16), the effect sizes for group differences in time spent markers were small to medium for training tasks (*r*s = .24-.49; Table 2) and large for assessment tasks (*r*s = .56-.73). Regarding post hoc tests, the effect sizes for the group differences were medium for age (*r* = .30; Table S2.4), small for race (Cramér's *V* = .16), very small for education and anxiety (*r*s = .08-.09), and very small or small for negative bias (*r*s = .08-.12).

When reviewing the unstandardized sizes of group differences (i.e., when comparing the median time spent by each group on a given task in Table 2), it is important to keep a few things in mind. First, the absolute difference between the groups' medians on a given task does not account for how long the task generally takes to complete; an absolute difference less than 1 min may seem small out of context, but such a difference can be large if the task generally does not take much time to complete. Important context is gained by considering the ratio of the groups' medians. For training tasks, the More Time Spent group spent 63.6% more time on the imagery practice exercise than did the Less Time Spent group, 53.0% more time on the anxiety imagery prime exercise, and 24.2% more time in terms of mean time per CBM-I scenario across sessions. For assessment tasks, the More Time Spent group spent 57% to 86% more time on the tasks than the Less Time Spent group (and 327% more time for the Credibility task).

Another important piece of context when comparing the median time spent by each group on a given task is what the task actually involves. Different tasks involve different amounts of textual instructions, different activities asked of the participant, and different numbers of items (e.g., see Table 1 for the number of items in each assessment task). Some tasks are very short and yet still have absolute group differences that are sizable given the tasks' brevity. For example, Anxiety Identity and Affect are each short, single-item tasks with minimal instructions, yet the absolute group differences in median time spent on these tasks are 7.8 and 4.8 seconds, respectively. We believe these are not trivial differences for single items (and, again, these differences correspond to the More Time Spent group's spending 68% and 62% more time on these items, respectively, and to large standardized effect sizes, *r*s = .60 and .57, respectively). Additionally, it is important to keep in mind the repeated nature of most of the tasks in Table 2 (e.g., a group difference of 2.7

seconds for mean time per CBM-I scenario equates to a difference of 9 min when scaled to the full set of 200 scenarios that participants are asked to complete across training sessions).

With respect to group differences in treatment outcomes, as stated in the Effect Size section of the Method (p. 14), we computed the standardized mean difference between engagement groups at Session 5 and at follow-up as growth modeling analysis (GMA) *d*, including GMA *d* adjusted for baseline differences (which is equivalent to Cohen's *d*). As we had reported in the Negative Interpretation Bias section of the Results (p. 18), for negative bias assessed by the Recognition Ratings measure (RR), the greater improvement for participants who generally spent less time yielded a small standardized mean difference between the groups at Session 5 (*d* adjusted = 0.27; Table 3). After such participants then lost some training gains during the follow-up phase, the standardized mean difference between groups at follow-up was negligible (*d* adjusted = -0.01). For bias assessed by the BBSIQ, after the groups improved comparably during the training phase but participants who spent less time then lost some training gains during the follow-up phase, the standardized mean difference between groups at follow-up was very small (*d* adjusted = -0.09).

Meaning of Differences in Behavioral Engagement

As for whether the behavioral engagement groups reflect different patterns of cognitive ability versus willingness/effort to engage, we would like to emphasize that by *behavioral engagement*, we (and prior literature) are referring to extent of use (i.e., number of tasks completed and time spent on tasks), which has also been referred to as *participation* or *physical energy investment* (Nahum-Shani et al., 2022). However, engagement, a multidimensional construct, involves not only such physical energy investment, but also simultaneous *cognitive energy investment* and *affective energy investment* (Nahum-Shani et al.). It is important to distinguish between these three dimensions, and, to the reviewer's point, one cannot infer cognitive and affective energy investments from only measures of physical energy investment. Given that, as we stated in the Background (p. 4), "most research has analyzed extent of use, which has been assessed with behavioral metrics (e.g., time spent online, completion rate, frequency of use)...[20, 18]", most prior studies also cannot infer users' subjective experiences (e.g., effort) from their measures of behavioral engagement. Clearly, more research is needed that measures a broader range of energy investments.

With the current data, by conducting post hoc tests of differences in demographic and clinical characteristics between our engagement groups, we attempted to shed more light on the potential meaning of our users' behavioral engagement patterns. These results led us to consider (p. 25) that, indeed, it may be "that people in the group that generally spent more time, who were significantly older (medium effect) and less educated (very small effect) than people in the group that spent less time, had slower processing speed[69] and lower reading comprehension.[70]" However, one of the takeaways of our paper, as we stated in the Future Work section (p. 26; and in the Abstract and Conclusion) is that "Given that engagement is multifaceted, future research…should assess affective and cognitive investments in addition to physical investments, going beyond the typical focus on physical investments (time spent and other behavioral metrics).[20, 18]" Beyond these general recommendations, our post hoc analyses can help guide future research efforts because they highlight which individual differences (e.g., age and education) are associated with differences in engagement patterns, which can help us determine

which candidate processes to measure. For example, as we note in the Future Work section (p. 26), "it would be helpful to directly measure some of the different possible explanations for increased time (e.g., distraction, slower processing speed, doubt about responses)."

To more clearly define behavioral engagement as extent of use, we now introduce the term in the following sentence of the Background (p. 4).

> Engagement in DMHIs is a multifaceted construct with at least two parts: (a) extent of program use (i.e., *behavioral engagement*), and (b) the user's subjective experience throughout the program,[21] which involves both cognitive and affective aspects.[22]

We have also further clarified that our engagement groups are behavioral engagement groups in the following sentences of the Objective, Discussion, and Conclusion.

> Objective (p. 5): The present study analyzed whether behavioral engagement subgroups, created using an exploratory cluster analysis from markers of task completion rate and time spent on training and assessment tasks, show differential improvement…

> Discussion (p. 23): This study analyzed differences in anxiety and interpretation bias outcomes for different behavioral engagement patterns in anxious community adults undergoing web-based CBM-I.

> Conclusion (p. 27): This study used an exploratory cluster analysis to create two behavioral engagement groups revealed by use metrics in a clinical trial of web-based CBM-I for anxiety.

As a side note, we are unclear what the reviewer means by their comment that "the training tasks in CBM-I are set one-time, fixed, and unrepeatable." However, given this comment, we would like to clarify that, in the present study, CBM-I was administered at each of five training sessions and that each session included a unique set of 40 CBM-I training scenarios. We have revised the sentences below in the Cognitive Bias Modification for Interpretation section (p. 7) accordingly:

> Each CBM-I session, designed to take about 15 min, consisted of a unique set of 40 ambiguous scenarios…Participants selected one or more letters to complete the word fragment, which increased in difficulty across the five sessions.

Study Implications

Regarding our study's implications, our study (a) illustrates the use of cluster analysis to analyze complex behavioral engagement patterns from a person-centered approach (as described on pp. 4-5). Our study also (b) found unexpected differences between clusters with respect to the common hypothesis that greater program use will yield better outcomes (see pp. 23-24). The unexpected differences in turn (c) highlight the challenge of using time spent metrics as markers of engagement and the need to consider cognitive and affective markers in addition to behavioral markers (see pp. 24-25). Finally, our study (d) shows the value of further characterizing the clusters on demographic and clinical characteristics, which can offer clues as to how the clusters

might have originated and why the clusters might have different (and in our case unexpected) treatment outcomes (see p. 25). For example, if we were to find that participants who generally spent more time (who tended to be older and less educated than participants who spent less time) had greater distractibility or lower reading comprehension, then clinically, we might shift the program's instructions or reading level for this group so they can optimize their outcomes.

To further highlight the value of our post hoc tests of cluster differences, we added the following sentence to the section Further Characterizing Engagement Groups (p. 25).

> Although the potential impacts of the group differences in age and education on engagement patterns and treatment outcomes noted above are speculative, they show the value of further characterizing the clusters for clues as to how the clusters might have originated and why the clusters might have some different outcomes.

We also added two sentences (underlined below) to summarize the engagement groups and their relative treatment outcomes in the Conclusion (p. 27), which now reads as follows.

> Understanding differences in treatment outcomes for different real-world patterns of engagement with DMHIs is key to clarifying how and for whom DMHIs are effective and to personalizing DMHIs. This study used an exploratory cluster analysis to create two behavioral engagement groups revealed by program use metrics in a clinical trial of a web-based CBM-I program for anxiety. Unexpectedly, the engagement groups (whose patterns ended up reflecting generally more vs. less time spent across tasks) did not significantly predict differential change in most outcomes. Moreover, participants who generally spent less time on the program (including both training and assessment tasks) improved in one outcome significantly more during the training phase (but had a significant loss in gains for that outcome by 2-month follow-up) than those who spent more time (and post hoc tests found were significantly older and slightly less educated). These unexpected group differences in outcomes highlight the challenge of using time spent metrics as markers of engagement and the need to consider cognitive and affective markers in addition to behavioral markers. Future research on complex engagement patterns in DMHIs holds promise for maximizing their potential to increase access to effective treatment for anxiety.

In summary, despite the clear, sophisticated and robust statistical analyses in this study. The ambiguous definition and measurement of "behavioural engagement", rationale and advantages of cluster analysis, and limitation in interpretation and (clinical) implications, will hamper the value of this manuscript.

Response: We thank the reviewer again for their feedback and hope our revisions have clarified our person-centered research question, the definition of behavioral engagement, the rationale for our cluster analysis and inclusion of engagement markers based on assessment tasks in addition to training tasks, and the interpretation and implications of our results.
