# Supplementary material for: Behavioral engagement patterns and psychosocial outcomes in web-based interpretation bias training for anxiety
Source: PLOS Digit Health. 2025 Jul 24;4(7):e0000945. doi: 10.1371/journal.pdig.0000945 (PMC12289040; doi:10.1371/journal.pdig.0000945)
Supplement: Attachment — Submitted filename: Response to Reviewers.pdf [file pdig.0000945.s003.pdf]

Response to Reviewers' Comments to the Authors

Reviewer 1

Points 1, 3, 4, and 6

The revisions made in response to the previous reviewers' feedback are commendable. These improvements have significantly enhanced the clarity and quality of the work, making its findings more effectively communicated.

Overall, this is a well-executed study with meaningful implications for enhancing online mental health treatments. It makes a significant contribution to the evolving field of digital mental health interventions.

The research is well-structured, methodologically robust, and provides valuable insights for clinicians, researchers, and developers of digital mental health programs.

This abstract is well-structured and clearly outlines the key objectives regarding behavioral engagement patterns and psychosocial outcomes in web-based interpretation bias training for anxiety.

Response: We appreciate the reviewer's kind words and acknowledgment of the clarity, rigor, and implications of our paper!

Point 2

The unexpected finding that lower-engagement users initially improved more in negative interpretation bias but later lost these gains is interesting, Do you have anything to provide a compelling theoretical explanation for this pattern

Response: We thank the reviewer for their interest in this finding. To better understand the two engagement groups, we conducted post hoc tests of group differences on demographic and other variables that did not enter into the cluster analysis. These tests revealed that people in the group that generally spent less time on the program were significantly younger and more educated than people in the group that spent more time. This raises the interesting (albeit unmeasured) possibility that people in the group that spent less (vs. more) time had faster processing speed (Salthouse, 2009) and higher reading comprehension (Caplan et al., 2011) on average, which would be consistent with this group's spending less time on both training and assessment tasks (as we observed). Moreover, faster processing speed and higher reading comprehension would likely make it easier for these participants to quickly learn the pattern presented in the training scenarios (i.e., that emotionally ambiguous situations tend to end in non-threatening ways). However, if these participants spent less time, it is possible that they were not thinking about the scenarios as deeply or imagining them as vividly as participants who spent more time, so those who spent less time might have been less likely to internalize the pattern and practice the skills in the long term, despite noticing this pattern in the short term (see Bjork, 1994; Mathews & Mackintosh, 2000; Menne-Lothmann et al., 2014). As a result, the initial learning for the Less

Time Spent group that occurred while the participants actively practiced assigning less negative interpretations during CBM-I may have eroded more quickly for this group (vs. the More Time Spent group) after that practice had ended.

We have added this potential theoretical explanation to the Further Characterizing Engagement Groups section of the Discussion (p. 25), which now reads:

> Post hoc tests revealed that the groups significantly differed on aspects other than time spent, providing potential insight into our findings for negative interpretation bias . . . Specifically, our post hoc tests raise the possibility that people in the group that generally spent more time on the program, who were significantly older (medium effect) and less educated (very small effect) than people in the group that spent less time, had slower processing speed[67] and lower reading comprehension.[68] These differences would likely add to their time (on both training and assessment tasks, as was observed) and potentially make it more difficult to quickly learn the pattern presented in the training scenarios (that ambiguous situations tend to end in non-threatening ways), whereas participants who spent less time may have more readily learned this pattern in the short term. However, once users who spent more time had improved, perhaps their gains were maintained at follow-up (unlike users who spent less time, who lost some gains) because of their greater physical investment of time during the training phase. In particular, if users who spent more time thought about the scenarios more deeply or imagined them more vividly, this elaborate processing could have facilitated longer-term recall (Bjork, 1994; Mathews & Mackintosh, 2000; Menne-Lothmann et al., 2014). By contrast, if the initial learning for users who spent less time was more superficial, it could have eroded more quickly after the active practice of assigning less negative interpretations during CBM-I had ended.

References Added:

Bjork, R. A. (1994). Memory and metamemory considerations in the training of human beings. In J. Metcalfe & A. P. Shimamura (Eds.), *Metacognition: Knowing about knowing* (pp. 185–205). MIT Press. https://psycnet.apa.org/record/1994-97967-009

Menne-Lothmann, C., Viechtbauer, W., Höhn, P., Kasanova, Z., Haller, S. P., Drukker, M., van Os, J., Wichers, M., & Lau, J. Y. F. (2014). How to boost positive interpretations? A meta-analysis of the effectiveness of cognitive bias modification for interpretation. *PLOS One, 9*(6). https://doi.org/10.1371/journal.pone.0100925

Point 5

The study does not sufficiently address potential confounding factors that could explain why participants who spent less time showed initial benefits but lost them at follow-up?

Response: We thank the reviewer again for their interest in this finding. In our response to their Point 2 above, we elaborated on potential reasons that people in the group that generally spent less time improved in negative interpretation bias (on one of two measures) significantly more during the training phase (vs. people in the group that spent more time) but then had a significant

loss in training gains for negative bias (on both measures) during the follow-up phase (whereas participants who spent more time had no significant change). Given that our post hoc tests found that the Less Time Spent group was significantly younger and more educated, we speculated that people in this group may have faster processing speed and higher reading comprehension, which could have made it easier for the people in this group to quickly learn the pattern presented in the scenarios. However, if the participants who spent less time did not process the scenarios as elaborately as participants who spent more time, then their initial learning during the training phase could have eroded more quickly during the follow-up phase.

In the Issues in Measuring Engagement section of the Discussion (pp. 24-25), we also discussed the challenges of the common interpretation of time spent as a marker of engagement. We noted that although time spent may indicate "physical energy investment (i.e., behavioral engagement or participation)", it "is a less clear indicator of behavioral investment than the markers used in some prior person-centered studies of engagement groups[17, 19, 66]." Moreover, we noted that "we do not know exactly how users were spending time, so distraction could have contributed to longer times." We also noted that engagement involves not only physical energy investment, but also "simultaneous cognitive (e.g., selective attention to the task) and affective (e.g., positive emotional reactions to and commitment to the task) energy investments."

Although our post hoc tests further characterizing the groups (e.g., on age and education, as we described above) provide clues as to how the groups might have originated and why the groups might have different outcomes (e.g., involving potential differences in processing speed; reading comprehension; and depth of processing the training scenarios, as we discussed above), we note in the paper that these ideas are speculative (p. 26). We are hesitant to overinterpret the finding that the Less Time Spent group improved more in negative bias during the training phase, as we found this for only one of two measures, but we have tried to offer some (speculative) possible explanations for the findings that could be tested in future research and also discuss the challenges of interpreting time spent metrics.

Finally, while we appreciate the opportunity to elaborate on potential reasons that the groups showed differential change in outcomes, we would like to note that, from the person-centered approach used in this paper, we do not consider actual or potential group differences as what the reviewer referred to as "potential confounding factors." The reason is that our research question did not seek to isolate the unique, independent, unconfounded average relation between time spent (as a 1-dimensional predictor) and outcomes (as might be the focus in a variable-centered approach). Rather, our research question concerned differences in outcomes for different participant clusters, where each cluster reflects a unique configuration, pattern, or set of values across all of the 17 engagement variables in a complex, multidimensional space. As we noted in the Further Characterizing Engagement Groups section (p. 25), in a person-centered approach it is valuable to consider any group differences on variables that did *not* enter into the cluster analysis, which we did in our post hoc tests for clues as to how the clusters might have originated and why the clusters might have some different outcomes. However, "this contrasts with a variable-centered approach, in which any correlates of a given engagement variable would represent potential confounds of the effect of the engagement variable on outcomes."

To the reviewer's point, as we partly discussed in our prior response letter (see Reviewer 4, Point 3, p. 8), a variable-centered approach seeking to isolate the unique, independent, unconfounded relation between an engagement variable and outcomes would absolutely face challenges if uncontrolled "third variables" (e.g., demographic or clinical characteristics) confounded the unique effect of the engagement variable on outcomes. However, in a person-centered approach, demographic, clinical, and other variables that did not enter into the cluster analysis provide a means of better understanding the clusters, which (by virtue of being partitions of a multidimensional space) are complex. As such, the reason we ran post hoc tests of cluster differences on demographic and clinical characteristics was not to rule out potential confounds of an engagement-outcome relationship, but rather to better understand the clusters, recognizing patterns of engagement as multifaceted and complex.

Point 7

Future interventions should incorporate cognitive markers (e.g., attention, cognitive load) and affective markers (e.g., motivation, task relevance). Future studies should also examine how these factors influence engagement and long-term outcomes.

Response: We agree that future research should assess markers of cognitive and affective investments in addition to physical investments, consistent with the idea that engagement is a multifaceted construct involving physical, cognitive, and affective energy investments. After introducing engagement as a "multifaceted construct" in the Introduction (p. 4), we expand on the three types of investments in the Issues in Measuring Engagement section of the Discussion (pp. 24-25):

> Although spending more time may reflect greater physical energy investment (i.e., behavioral engagement or participation)...engagement involves simultaneous cognitive (e.g., selective attention to the task) and affective (e.g., positive emotional reactions to and commitment to the task) energy investments as well.[22]

In the Future Work section (p. 27), we also suggest that future research assess all three investments. We have added examples of cognitive and affective investments to assess, and noted that future research should study how multifaceted patterns of such investments affect outcomes (maintaining our person-centered focus on patterns).

> Given that engagement is multifaceted, future research on engagement in DMHIs (for both CBM-I and other interventions) should go beyond the typical focus on physical energy investments (time spent and other behavioral metrics).[20, 18] Future work could assess affective investments (e.g., perceiving tasks as self-relevant, feeling motivated to do tasks) and cognitive investments (e.g., selectively attending to tasks, using mental resources to meet task demands) in addition to physical investments,[22] and study how multifaceted patterns of these investments influence outcomes . . . It would also be helpful to directly measure some of the different possible reasons for increased time (e.g., distraction, slower processing speed, doubt about responses).

Reviewer 2

Point 1

I appreciate the valuable contribution this study makes to understanding engagement patterns in digital mental health interventions, particularly in the context of web-based cognitive bias modification for interpretation (CBM-I) in anxious adults. One of the most important insights from this research is that measuring engagement purely in terms of time spent may be insufficient for optimizing DMHI effectiveness, which has significant implications for intervention design and evaluation.

Response: We thank the reviewer for their feedback and recognition of our paper's implications.

Point 2

The authors have also done an excellent job in addressing the reviewers' previous comments, providing thorough explanations and clarifications that have strengthened the manuscript. However, I recommend that they acknowledge the limitations of their sample, as it consists of self-selected anxious adults in an online setting. This may affect the generalizability of the findings, particularly for clinical populations or individuals with lower digital literacy.

Response: We appreciate this suggestion. We have added the following underlined text to the Limitations section of the Discussion (p. 26):

> Third, our sample was demographically homogeneous, and we may have introduced sampling bias by excluding participants who were assigned to receive supplemental telecoaching. Further, although all participants had at least moderate anxiety symptoms at baseline (and on average had *marked* [Bragdon et al., 2016] total OASIS scores [11.55; Table S2.2], surpassing the cutoff of 8 for probable diagnosis [Campbell-Sills et al., 2009; Norman et al., 2011; Moore et al., 2015], and *extremely severe* [Lovibond & Lovibond, 1995] total DASS-21-AS scores [11.27, or 22.54 on the DASS-42-AS]), our results may not generalize to other populations (e.g., those defined by clinical diagnoses; those who would not seek web-based CBM-I).

References Added:

Bragdon, L. B., Diefenbach, G. J., Hannan, S., & Tolin, D. F. (2016). Psychometric properties of the Overall Anxiety Severity and Impairment Scale (OASIS) among psychiatric outpatients. *Journal of Affective Disorders, 201*, 112–115. https://doi.org/10.1016/j.jad.2016.05.005

Campbell-Sills, L., Norman, S. B., Craske, M. G., Sullivan, G., Lang, A. J., Chavira, D. A., Bystritsky, A., Sherbourne, C., Roy-Byrne, P., & Stein, M. B. (2009). Validation of a brief measure of anxiety-related severity and impairment: The Overall Anxiety Severity and Impairment Scale (OASIS). *Journal of Affective Disorders, 112*(1–3), 92–101. https://doi.org/10.1016/j.jad.2008.03.014

Lovibond, S.H. & Lovibond, P.F. (1995). *Manual for the Depression Anxiety Stress Scales* (2nd Ed.). Sydney: Psychology Foundation. ISBN 7334-1423-0.

Moore, S. A., Welch, S. S., Michonski, J., Poquiz, J., Osborne, T. L., Sayrs, J., & Spanos, A. (2015). Psychometric evaluation of the Overall Anxiety Severity and Impairment Scale (OASIS) in individuals seeking outpatient specialty treatment for anxiety-related disorders. *Journal of Affective Disorders, 175*, 463–470. https://doi.org/10.1016/j.jad.2015.01.041

Norman, S. B., Campbell-Sills, L., Hitchcock, C. A., Sullivan, S., Rochlin, A., Wilkins, K. C., & Stein, M. B. (2011). Psychometrics of a brief measure of anxiety to detect severity and impairment: The Overall Anxiety Severity and Impairment Scale (OASIS). *Journal of Psychiatric Research, 45*(2), 262–268. https://doi.org/10.1016/j.jpsychires.2010.06.011

Point 3

Additionally, while the study convincingly argues that time spent is an inadequate engagement marker, it would be helpful to suggest concrete strategies for improving CBM-I interventions. For instance, should engagement be adaptive, with task length adjusting based on user progress? Addressing these aspects would further enhance the manuscript's clarity and practical relevance.

Response: We thank the reviewer for this idea. We have added these strategies for improving research on engagement in DMHIs to the future directions we had listed in the Future Work section of the Discussion (p. 27): (a) more specific examples of affective and cognitive energy investments to assess in addition to physical investments, (b) a suggestion to further characterize clusters on variables that did not enter into the cluster analysis (as we did in our post hoc tests), and (c) a suggestion to administer training and assessment tasks in parallel (vs. in series). We have also (d) suggested techniques that future CBM-I programs might include during, at the end, and after training if, as we speculated above (see our response to Reviewer 1 Point 2), users who generally spent less time lost some training gains because they processed the training scenarios less elaborately than users who generally spent more time.

> Given that engagement is multifaceted, future research on engagement in DMHIs (for both CBM-I and other interventions) should go beyond the typical focus on physical investments (time spent and other behavioral metrics).[20, 18] Future work could assess affective investments (e.g., perceiving tasks as self-relevant, feeling motivated to do tasks) and cognitive investments (e.g., selectively attending to tasks, using mental resources to meet task demands), in addition to physical investments,[22] and study how multifaceted patterns of these investments influence outcomes. Another potentially interesting physical marker is time elapsed between sessions (which we did not include in our cluster analysis because, due to attrition, only 399 participants had the data needed to compute such a variable; see Table S2.11). It would also be helpful to directly measure some of the different possible reasons for increased time (e.g., distraction, slower processing speed, doubt about responses). If, as we speculated, users who generally spent less time lost some training gains because they processed the training scenarios less elaborately, future CBM-I programs might include techniques to encourage deeper processing during training (e.g., exercises that apply insights from training to users' own experiences), consolidate gains at end of training (e.g., relapse-prevention exercises), and

facilitate skill application after training (e.g., skill reminders, booster sessions). Future work could also identify individual engagement markers that predict outcomes before performing clustering on only the significant predictors.[30, 17] To investigate variability in engagement over time, growth mixture models could identify unobserved subgroups from repeated measures of engagement.[69, 70] The present study also highlights the value of further characterizing engagement patterns on variables that did not enter into the cluster analysis (e.g., demographic characteristics, symptom severity), a strategy we suggest for future research. Finally, to analyze separate completion rates for training versus assessment tasks, future work should administer these tasks in parallel, allowing users who stop training to continue assessments.

Reviewer 3

Thank you for the opportunity to review this manuscript. It presents an interesting analysis and take on engagement with a web-based training program for anxiety. I have very few comments, I think it is very well written and clearly explained and really only have a couple of thoughts that I felt were missing from the discussion.

Response: We are grateful for the reviewer's interest in our paper and for their comments.

Point 1

I understand your rationale for performing your analysis this way and using the clusters to explore complex patterns of engagement. After reading the manuscript, I wondered whether this is the best way to explore engagement? Given no differences and the difficulties interpreting time spent, on reflection would task completion actually have given a better sense of how much people actually worked through the content? Since you don't seem to have done any post-hoc analysis looking at this, or refer to other work that did do this with this data, I felt this could be discussed in the discussion.

Response: We appreciate the reviewer's suggestion. We have added further discussion of the value of examining a variety of metrics that could indicate behavioral investment (including task completion rates) to the Issues in Measuring Engagement section (pp. 24-25).

> Although spending more time may reflect greater physical energy investment (i.e., behavioral engagement or participation), time spent is a less clear indicator of behavioral investment than the markers used in some prior person-centered studies of engagement groups[17, 19, 66], such as task completion rate (which was balanced between the groups in our study) and frequency of using the program (which was constrained in our study given that users could not repeat sessions). Effective engagement is investment directed at the focal tasks in the DMHI that are thought to lead to change in outcomes (e.g., targeted mechanisms and downstream symptoms).[22] . . . However, we do not know exactly how users were spending time . . . Moreover, engagement involves simultaneous cognitive (e.g., selective attention to the task) and affective (e.g., positive emotional reactions to and commitment to the task) energy investments as well.[22] Thus, although time spent may offer some insight into the process by which users complete tasks, it is

> critical to understand the actual behaviors and cognitive and affective experiences that underlie users' time spent, while also examining a variety of other markers of behavioral investment (e.g., task completion rates) and its experiential qualities.

Although task completion rate is a valuable behavioral investment marker, we do not think it would be advisable to analyze *only* completion rate (which, e.g., lacks information on the process by which users complete tasks). Moreover, if we had analyzed only completion rate, we likely would have obtained clusters that we would have been unable to compare on treatment outcomes. Specifically, we likely would have found two clusters with low versus high task completion rates and likely would have had insufficient outcome data for the low completion rate group.[1] The low completion rate group would have less outcome data than the high completion rate group because the training and assessment tasks were administered in series (vs. parallel), meaning that if participants did not complete a training or assessment task they were unable to complete any subsequent training and assessment tasks. (We already noted a related limitation on page 26 that prevented "analysis of completion of training tasks separate from assessment tasks," and we have now added a suggestion on page 27 that "to analyze separate completion rates for training versus assessment tasks, future work should administer these tasks in parallel, allowing users who stop training to continue assessments.")

Point 2

Similarly although you talk about the differences between groups which is very interesting, you don't do any post-hoc analysis controlling for these. Again I felt maybe this was missing in the discussion, whether these are worth further exploration in analysis, how they might be impacting outcomes.

Response: We appreciate the reviewer's attention to this. Although statistically controlling for the demographic and clinical characteristics that we compared the groups on post hoc might be advisable in a variable-centered approach to research, in our present person-centered approach controlling for these characteristics is not advisable.

As we noted in our response to Reviewer 1 Point 5 above, our research question did not concern the unique, independent, unconfounded relationship between time spent (as a 1-dimensional predictor) and outcomes (as might be the focus in a variable-centered approach). Rather, our research question concerned differences in outcomes for different participant clusters, where each cluster reflects a unique configuration, pattern, or set of values across all of the 17 engagement variables in a complex, multidimensional space. As a result, controlling for the demographic and clinical characteristics is not advisable in our person-centered approach.

---

[1] This is suggested by the bimodal distribution of task completion rate (Figure S3.4), and, indeed, we found two low vs. high completion rate groups when we included a smaller number of time-related training and assessment markers in our initial cluster analysis. However, as we note in Section S1.6.1 of the supplement (p. 5), because most participants in the low completion rate group of our initial cluster analysis did not finish the first training session and thus had assessment data only at baseline, the imputation model used to handle missing outcome data at later time points did not converge. This would have prevented appropriate missing data handling in the multilevel models used to analyze differences between engagement groups in treatment outcomes.

As discussed earlier (see Reviewer 4, Point 3, p. 8, in our prior response letter), the reason we ran post hoc tests of cluster differences on demographic and clinical characteristics was not to rule out potential confounds of an engagement-outcome relationship, but to better understand the clusters, recognizing patterns of engagement as multifaceted and complex. Thus, although it would technically be possible to control for such characteristics (e.g., age, education) by entering them as covariates in our multilevel models testing the effect of engagement group on treatment outcomes, doing so would defeat the purpose of better understanding the engagement groups, as this would remove relevant information about the actual composition of the two groups. From a person-centered perspective, such demographic and clinical differences are inherent to the actual clusters and relevant to understanding their patterns, not nuisances to be statistically controlled.

To reiterate our person-centered approach and clarify how such group differences are interpreted in this approach, we had added the following text to the Further Characterizing Engagement Groups section of the Discussion (p. 25).

> Post hoc tests revealed that the groups significantly differed on aspects other than time spent, providing potential insight into our findings for negative interpretation bias. Notably, in our person-centered approach, any engagement group differences on variables that did not enter into the cluster analysis can help inform interpretation of the groups' engagement patterns. (This contrasts with a variable-centered approach, in which any correlates of a given engagement variable would represent potential confounds of the effect of the engagement variable on outcomes.)

As we noted in our response to Reviewer 1 Point 2 above, in the Further Characterizing Engagement Groups section (p. 25) we now expand on how these characteristics of the groups might be impacting the groups' differential outcomes for negative interpretation bias. This is an example of trying to understand the actual behaviors and cognitive and affective experiences that underlie users' time spent to obtain a fuller picture of users' complex, multifaceted engagement patterns and to consider how those engagement patterns could have different outcomes (vs. isolating the unique effect of an engagement variable on outcomes, which was not our goal).

Additional Changes

As requested during pre-review of this revision, we also (a) removed all footnotes, incorporating their content into the main body text of the manuscript (and moving part of one footnote to a new supplemental Section S1.11 Post Hoc Duda-Hart Test) and (b) listed the supplemental sections in a new Supporting Information Legends section of the manuscript (p. 41).
